# Genomic diversity of the Japanese wheat core collection and selection of alleles for agronomic traits in the breeding process

**DOI:** 10.1270/jsbbs.23064

**Published:** 2024-06-25

**Authors:** Nobuyuki Mizuno, Fuminori Kobayashi, Takumi Morita, Hirokazu Handa

**Affiliations:** 1 Institute of Crop Science, National Agriculture and Food Research Organization, 2-1-2 Kannondai, Tsukuba, Ibaraki 305-8518, Japan; 2 Graduate School of Life and Environmental Sciences, Kyoto Prefectural University, 1-5 Shimogamo-hangicho, Sakyo, Kyoto 606-8522, Japan

**Keywords:** Japanese core collection, genetic diversity, genome-wide association, selected agronomic genes, breeding history, wheat

## Abstract

Combining high-throughput genotyping data with the latest wheat genomic information provided more detailed information on the genetic diversity of the Japanese wheat core collection (JWC). Analysis of genomic population structure divided the JWC accessions into three populations: northeast Japan accessions, native and southwest Japan accessions, and modern accessions showing mixed breeding patterns. This indicates that Japanese wheat varieties have a background of native genomes from southwest Japan incorporating valuable genes from various exotic lines, which is supported by the history of Japanese wheat breeding. Association analyses of several agronomic traits have revealed how genes or alleles have been selected in Japanese wheat breeding and how they differ from those in other regions of the world. This analysis of the JWC collection is expected to contribute not only to the elucidation of genetic diversity in Japanese wheat accessions but also to future wheat breeding by providing a new genetic resource.

## Introduction

Bread wheat (*Triticum aestivum* L.) is one of the most important cereals, grown in 219 million hectares worldwide, with 761 million tons harvested in 2020. Wheat contributes approximately 20% of the total calories and protein required by humans and provides more protein than any other food source, including animal products (FAOSTAT 2021). Wheat is becoming increasingly important as the human population continues to grow. To meet this population growth, the demand for wheat should increase by 1.7% annually, reaching 1 billion tons by 2050; however, the average annual increase in wheat revenue is currently only 1% (Tadesse *et al.* 2019). Various breeding strategies have been implemented using conventional and molecular breeding approaches. It is essential to use a wide range of genomic resources to determine the genetic diversity of wheat and to determine the best allele combinations for developing improved varieties.

Since the sequencing of the world’s first wheat reference genome in 2018 (IWGSC 2018), various efforts have been made to understand the genetic diversity of wheat at the DNA level. Using 4,506 wheat accessions from 108 countries, including cultivars with a significant impact on wheat breeding, such as the Green Revolution, the dissemination and differentiation of wheat have been revealed on a haplotype basis (Balfourier *et al.* 2019). This showed that the split into two gene pools, European and Asian, occurred during the initial spread of bread wheat from its origin, the Fertile Crescent, to the rest of the world. Subsequently, wheat was further differentiated into eight subpopulations. To further reveal the details of the genomic diversity of wheat breeding and research programs spanning several continents, a pangenome analysis was carried out, in which the reference-level assemblies for ten wheat accessions, including cultivars, breeding lines, and a spelt landrace were completed, in addition to five wheat genome assemblies at the scaffold level (Walkowiak *et al.* 2020). These pangenomic analyses revealed a significant bias in the use of genetic resources in modern wheat breeding. In particular, the results showed that the genetic diversity of wheat originating from Asia is largely underutilized in many modern breeding programs worldwide (Balfourier *et al.* 2019). Sequence data also confirmed that Asian germplasms from Japan and China, for example, differ significantly from those in the rest of the world (Shimizu *et al.* 2021, Walkowiak *et al.* 2020). On the other hand, it is well known that Asian germplasms contain important genes, such as *Rht* dwarfing genes (Hedden 2003) and disease resistance genes, such as *Fhb1* (Niwa *et al.* 2018, Rawat *et al.* 2016), which are essential for breeding modern varieties. These Asian breeding groups may be valuable reservoirs for novel alleles and useful genes.

Japan is located to the east of the Eurasian continent, far from West Asia, where wheat originated, and its climate is very different from that of its origin. Japanese wheat accessions are thought to have evolved adaptations to the unique environmental conditions of high temperature and humidity (Oda 2015). In addition, the need to avoid preharvest sprouting during the harvest period and the constraints imposed by the double-cropping system with rice led to a marked increase in early maturity. Short culms also characterize Japanese cultivars owing to semidwarf genes, such as *Rht-1* in Norin 10, which became well known worldwide during the Green Revolution (Hedden 2003, Peng *et al.* 1999). Thus, Japanese cultivars are distinctive from other cultivars worldwide. The differences between wheat accessions in Japan and those in other regions at the whole-genome sequence level will reveal the evolution of wheat adaptations to the environment and provide a basis for breeding wheat in response to climate change and other factors. To this end, we recently decoded the whole-genome sequence of a Japanese wheat cultivar, Norin 61, at the reference level, which is a representative Japanese cultivar characterized by broad adaptation with environmental robustness (Shimizu *et al.* 2021). The genome sequence revealed several unique genetic variations of Norin 61, the *Fhb1* locus for *Fusarium* head blight resistance, *Ppd-D1a* for early flowering, *Glu-D1f* for Asian noodle quality, and *Rht-D1b* for semidwarfism, as expected. Following the genome sequencing of Chinese Spring (IWGSC 2018), Norin 61 genome decoding has comprehensively characterized the underexplored bread wheat diversity in Asia.

To expand our knowledge of the genetic diversity of Japanese wheat accessions and to use them in breeding programs efficiently, we analyzed the Japanese wheat core collection (JWC) selected by the National Agriculture and Food Research Organization (NARO) (Kojima *et al.* 2017). Previously, we performed genome-wide single nucleotide polymorphism (SNP) genotyping using the genotyping-by-sequencing (GBS) method to reveal the molecular basis of traits in the JWC. Phylogenetic trees and population structure analyses clarified the genetic diversity and mutual relationships of each JWC accession, and a preliminary genome-wide association study (GWAS) was conducted (Kobayashi *et al.* 2016). However, due to the lack of reference genome sequences for wheat at that time, the quality and accuracy of the analyses were not sufficient. In this study, we detected genome-wide SNPs using the data previously obtained by GBS (Kobayashi *et al.* 2016) and newly developed data from ddRAD-Seq and amplicon-seq, in combination with the reference sequence data of the wheat genome (IWGSC 2018, Zhu *et al.* 2021), and performed genome-wide analyses, including GWAS, on eight agronomic traits measured under field cultivation. The results reveal the genetic diversity and breeding impact of the JWC.

## Materials and Methods

### Plant materials and phenotypic observation

The NARO Japanese wheat core collection (JWC) maintained at the GeneBank Plant Section was used (https://www.gene.affrc.go.jp/databases-core_collections_jw_en.php), which consists of 96 accessions, including 75 breeder lines, 11 landraces and 9 unknown lines, and the cv. Chinese Spring (CS) (Supplemental Table 1). To show the phenotypic diversity of the collection, we evaluated the days to heading (DH), culm length (CL), spike length (SL), spikelet number per spike (SNS), spike density (SD; SNS divided by SL), grain number per spike (GNS), grain number per spikelet (GNSL), and thousand-kernel weight (TKW). Phenotypic evaluation was carried out in the experimental field of the Institute of Crop Science, NARO (Tsukuba, Japan), under natural field conditions for five years (from 2016 to 2020). The sowing date was the day of suitable conditions for sowing between October 20th and November 8th of each season. Each experimental plot consisted of a single 90-cm-long row with 20 cm spaces between plants. Three individuals were grown per plot. Harvesting took place between late June and early July of each season. Three replicates were measured for each trait, and the mean values were used in the following analysis. Phenotypic variation analysis, normal distribution adherence, and Pearson’s correlation analysis of the JWC accessions were performed and tested using Microsoft Excel for Mac (ver. 16). Broad-sense heritability (*h*^2^) was estimated following the method described by Collin *et al.* (2019).

### Genotyping data

The sequence reads for all 96 accessions in the JWC using the genotyping-by-sequencing method has been reported previously (Kobayashi *et al.* 2016). We also used the amplicon-seq technique developed by Ishikawa *et al.* (2018) for SNP calling in the JWC accessions. Furthermore, we developed SNP markers by ddRAD-Seq. Genomic DNAs were digested with *Bgl*II and *Eco*RI, ligated with Y-shaped adaptors, amplified by PCR with KAPA HiFi HS ReadyMix (KAPA BIOSYSTEMS, Roche, Basel, Switzerland) and size-selected with E Gel Size Select (Life Technologies, CA, USA). Approximately 450 bp library fragments were retrieved. Further details of the library preparation method were described in a previous study (Sakaguchi *et al.* 2015). Sequencing was performed with the paired-end 151 bp mode of HiSeqX (Illumina, CA, USA). Low-quality reads and adaptors were trimmed using Cutadapt (v4.2) with --overlap 10 --minimum-length 51 --quality-cutoff 20 options (Martin 2011). The trimmed paired-end reads from three methods were respectively mapped against the wheat reference genome (IWGSC RefSeq v2.1, Zhu *et al.* 2021) using BWA-mem (v0.7.15) (Li and Durbin 2009), and multiple mapped reads were filtered using elPrep (v5.1.3) (Herzeel *et al.* 2021). Variants were detected using FreeBayes (v1.3.5) with the -C 2 -u -p 2 -F 0.2 options (https://github.com/ekg/freebayes). Heterozygous variants were converted to missing data using TASSEL 5 (Bradbury *et al.* 2007). We removed the markers with more than 50% missing data, a depth of less than five, a mapping quality of less than 20, an allele number of 3 or more, and a minor allele frequency (MAF) less than 5%. In addition, we used BEAGLE v3.3.2 (Browning and Browning 2009) to impute the data by identifying the haplotype cluster based on the non-missing genotypes. SNP data from the three methods were merged into one file. Then we retained genotype data from one method with the lowest missing rates before imputation using BEAGLE for the commonly detected sites by two or three methods. The SNP data for the JWC accessions can be accessed through Tasuke+ (https://wheat.dna.affrc.go.jp/).

Genotyping of *Rht-B1* and *Rht-D1* was conducted using PCR-based markers developed by Ellis *et al.* (2002). Primer sequences for detecting each allele are summarized in Supplemental Table 2. Each marker is dominant, but since they developed markers for both alleles it is possible to track every genotype combination. For accessions that could not be genotyped by PCR analysis, we cited the genotypes determined by Kojima *et al.* (2017).

### Population structure analysis

We used ADMIXTURE v1.3.0 (Alexander *et al.* 2009) to investigate the population structure of the 96 accessions in the JWC. Ten ADMIXTURE analysis runs were performed for each K value using different random seeds. The best run was selected based on the highest log-likelihood value. The results of the ADMIXTURE analysis were visualized using pong v1.5 (Behr *et al.* 2016). We performed principal component analysis (PCA) based on covariance using TASSEL 5 (Bradbury *et al.* 2007). We then constructed a maximum likelihood (ML) tree using RAxML-NG (Kozlov *et al.* 2019) with the “GTR + G + ASC_LEWIS” model and the bs-trees option of 1000. The best ML tree was rooted using midpoint rooting and visualized using FigTree 1.4.4 (http://tree.bio.ed.ac.uk/software/figtree/). We calculated the Weir and Cockerham mean *F*-statistics (Fst; Weir and Cockerham 1984) using the VCFtools 0.1.13 (Danecek *et al.* 2011).

### Statistical analyses

Statistically significant differences between phenotypic groups were determined using Tukey–Kramer’s HSD test in R software (*p* < 0.05). We generated scatter plots and box plots using the ggplot2 package in R software (R Core Team 2020). Regression lines in scatter plots were added with the “lm” method (Wickham 2016). We performed PCA of the phenotypic data using the prcomp function implemented in the ggbiplot package of R software with the “var.axes” and “covariance biplot” options. We constructed heatmaps using the heatmap2 package with the Ward D2 clustering method in R software.

### Mapping with GWAS

Marker–trait association tests were performed in TASSEL version 5.0 using the mixed linear model (Zhang *et al.* 2010) for eight traits with five years of replication, as mentioned above. We calculated the principal components using the pipeline implemented in TASSEL; the first two principal components were used as fixed effects. We used the kinship matrix among individuals estimated using the centered-IBS method (Endelman and Jannink 2012) as the covariance matrix for the random genotypic effects in the mixed linear model. We evaluated the magnitude of the marker–trait association (MTA) effects using the *R*^2^ value and determined the significance of marker associations with phenotypic traits using the Bonferroni method for multiple testing correction at an α level of 0.05 (*p* = 0.05/30,217 = 1.655 × 10^–6^, or –log_10_ (*p* value) = 5.781). We generated Manhattan plots based on the association mapping results with CMplot (Yin *et al.* 2021).

## Results

### SNP marker evaluation and genome-wide analysis of genetic diversity

Mapping previously obtained SNPs by the GBS method (Kobayashi *et al.* 2016), amplicon-seq and ddRAD-Seq (in this study) against the IWGSC RefSeq v2.1 (https://wheat-urgi.versailles.inra.fr/Seq-Repository/Assemblies, Zhu *et al.* 2021) resulted in the successful mapping of 30,217 SNPs after excluding those without missing data of less than 50%, a minor allele frequency less than 5%, and imputation. Mapping statistics in three methods and information regarding the number of markers and marker density for each chromosome were summarized in Supplemental Tables 3 and 4. The average marker density for the entire genome was one marker per 482 kbp. Chromosome 3B contained the most markers (3,362) and showed the highest marker density (one marker per 253 kbp). On the other hand, chromosome 4D contained the fewest markers (131) and had the lowest marker density (one marker per 3.957 Mbp). All SNP information for the JWC accessions has been compiled in a genome browser form using Tasuke+ (Kumagai *et al.* 2019) and can be freely accessed at the following URL: https://wheat.dna.affrc.go.jp/.

**Fig. 1.**
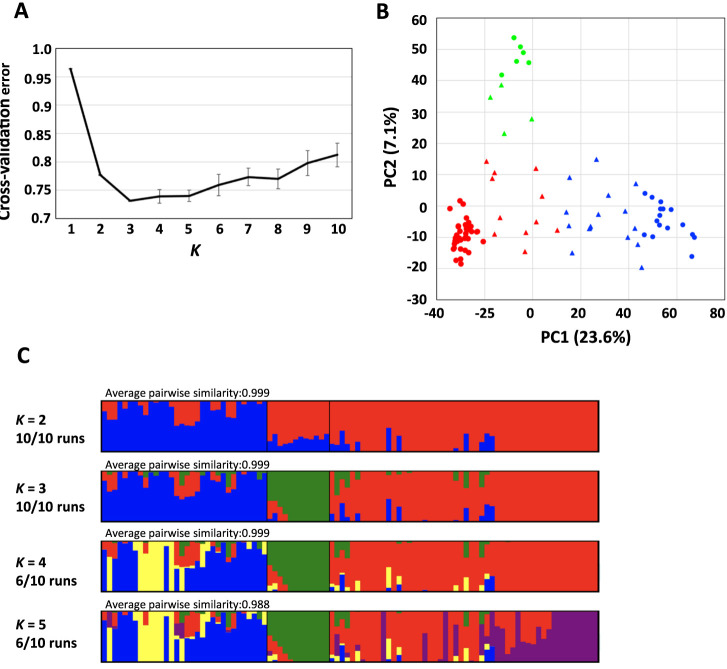
Population differentiation of the 96 JWC accessions. Cross-validation errors of ancestral population assignment for different numbers of clusters by ADMIXTURE (*K* = 1–10) (A). Graph of the first two axes (PC1 and PC2) from principal component analysis (PCA) (B). Population structure of 96 wheat accessions inferred using ADMIXTURE (*K* = 2–5) (C). Representative runs of ADMIXTURE were visualized using pong v1.5. The proportion of variance explained by each component is given in parentheses along each axis. Blue, green and red indicate the accessions belonging to populations I, II, and III, respectively. Triangles show the admixed accessions (estimated admixture proportion of <0.9).

We analyzed population structure using ADMIXTURE and principal component analysis (PCA) (Fig. 1, Supplemental Fig. 1). The cross-validation error in ADMIXTURE analysis decreased until *K* = 3 (Fig. 1A). It was almost flat from *K* = 3 to *K* = 5 and gradually increased from *K* = 6. We evaluated population structure using PCA (Fig. 1B). The percentages of variation explained by the first and second principal components were 23.6% and 7.1%, respectively. The PCA plot with the accessions color-coded according to *K* = 3 by ADMIXTURE analysis indicated that the number of clusters at *K* = 3 was suitable for the 96 JWC accessions (Fig. 1C). Populations I, II, and III contained 32, 12, and 52 accessions, respectively. Accessions identified as belonging to population I and population III clustered separately on both ends of the PC1 axis, corresponding to the accessions from the Hokkaido area and classical accessions from mainland Japan. The PC2 axis was divided into populations III and II, which were composed of modern cultivars. The accessions positioned near the boundaries of each population were estimated to be admixed (admixture proportion of <0.9). We also assessed population differentiation using Weir and Cockerham’s Fst values, which indicate population differentiation by genetic structure; a high FST means high differentiation between populations. The Fst values for all chromosomes among the three populations were high (>0.2, Supplemental Table 5).

### Phenotypic variations

The results for the measurements of the eight agronomic traits in the JWC accessions are summarized in Table 1. ANOVA showed significant differences (*p* < 0.01) among accessions for all traits examined over the five years. The broad-sense heritability (*h*^2^) of the eight traits over five years ranged from 71.3 to 90.0%, with the minimum for GNSL and the maximum for CL. The *h*^2^ values of the traits related to general growth and morphology, such as DH, CL, and SL, were high, whereas the *h*^2^ values for spike structure traits such as SNS, SD, GNS, GNSL, and TKW were relatively low. These results indicate that these traits are affected differently by the environment. The coefficients of variation (CVs) were also different for each trait but did not differ significantly over the years. In summary, although there are differences in traits, these results suggest that genetic factors play an essential role in the phenotypic variation of each trait in the JWC accessions and that these quantitative traits are under typical polygenic control.

The genetic correlation coefficients among the traits studied using Pearson’s approach are shown in Table 2. The general growth and morphological traits, DH, CL, and SL, showed significant positive correlations with each other. An association of spikelet number per spike (SNS) with these three traits was observed, but the significance levels were different among the traits (*p* < 0.001 for DH and CL and *p* < 0.05 for SL). The correlations between most traits related to spike structure, such as SD, GNS, GNSL, and TKW, varied in significance and direction, and a consistent trend was not observed. Furthermore, SL was significantly correlated with the other seven traits examined in this study, suggesting that SL interacts significantly with other traits.

**Fig. 2.**
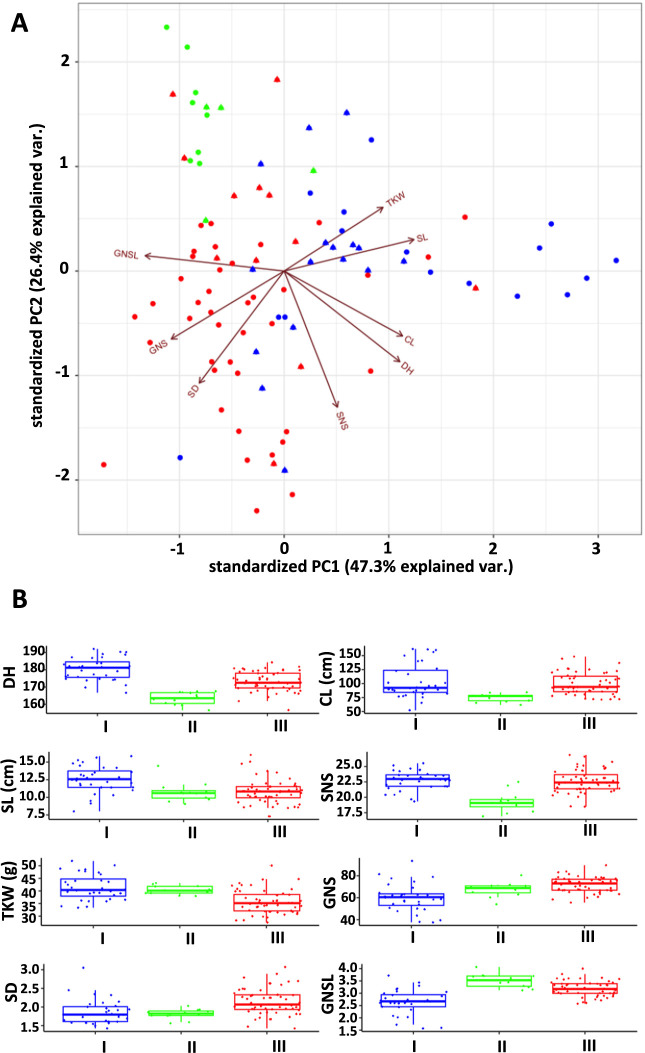
Phenotypic variation among populations of the JWC accessions. Scatterplot of the first two components (PC1 and PC2) from the principal component analysis (PCA) based on data for the phenotypes of eight traits in 96 JWC accessions (A). Blue, green, and red indicate the accessions belonging to populations I, II and III, respectively. Triangles show the admixed accessions (estimated admixture proportion of <0.9). Length of the arrows depicts the strength of the contribution of the feature in that direction. Comparison of eight traits among three populations (B). Mean values with the same letters are not significantly different (*p* > 0.05) (Tukey–Kramer’s HSD test). DH, days to heading; CL, culm length; SL, spike length; SNS, spikelet number per spike; TKW, thousand-kernel weight; GNS, grain number per spike; SD, spike density; GNSL, grain number per spikelet.

To examine the relationship between population structure and phenotypic diversity, we performed PCA using the phenotypic trait data. PC1 and PC2 accounted for 47.3 and 26.4% of the total phenotypic variance, respectively. The PCA plot exhibited partially overlapping distributions for the three populations (Fig. 2A); PC1 roughly separated population I from the others, whereas PC2 separated the accessions into populations II and III. Similar to the PCA plot based on genotype data, the phenotypic plot showed that admixed accessions were often located on the boundaries of each population.

**Fig. 3.**
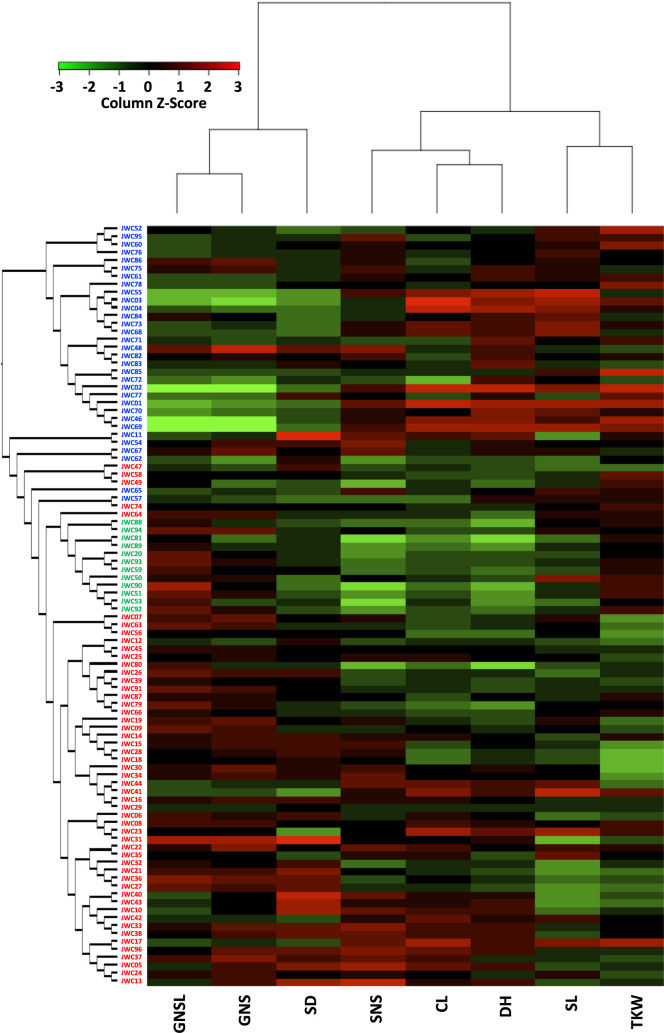
Heatmap of the eight phenotypic traits in the JWC accessions. The columns of the heatmap are ordered based on the maximum likelihood phylogenetic tree, which is shown on the left with its branch lengths transformed to be proportional. GNSL, grain number per spikelet; GNS, grain number per spike; SD, spike density; SNS, spikelet number per spike; CL, culm length; DH, days to heading; SL, spike length; TKW, thousand-kernel weight.

DH and GNSL were significantly different among the three populations, and the other six traits were also significantly different in at least one comparison between populations (Fig. 2B). Later heading, higher CL, lower GNS, and lower GNSL were the characteristics that separated population I from the other populations. The accessions belonging to population II generally showed significantly earlier heading, lower CL, lower SNS, and higher GNSL than those of the other two populations. In contrast, accessions belonging to population III exhibited significantly lower TKW, higher SD, and higher GNS. In Fig. 3, combining the ML tree and the heatmap based on the phenotypes offers a visual confirmation of the separation of JWC accessions, revealing a close association between the phylogenetic relationship and phenotypic variances of the eight traits. However, we found phenotypic differences between genetically similar accessions (e.g., JWC62 and JWC67, and JWC17 and JWC96).

### Detection of MTAs for spike length and culm length by GWAS

We performed marker–trait association analyses using the SNP dataset above to identify the gene loci responsible for the eight quantitative traits addressed here. We detected 220 MTAs with *p* values ≤0.001 for seven traits over multiple years (Supplemental Tables 6–12). No MTAs for SNS were detected over multiple years. We did not identify MTAs for traits over the significance level of 5% after Bonferroni multiple test correction (*p* = 1.655 × 10^–6^).

**Fig. 4.**
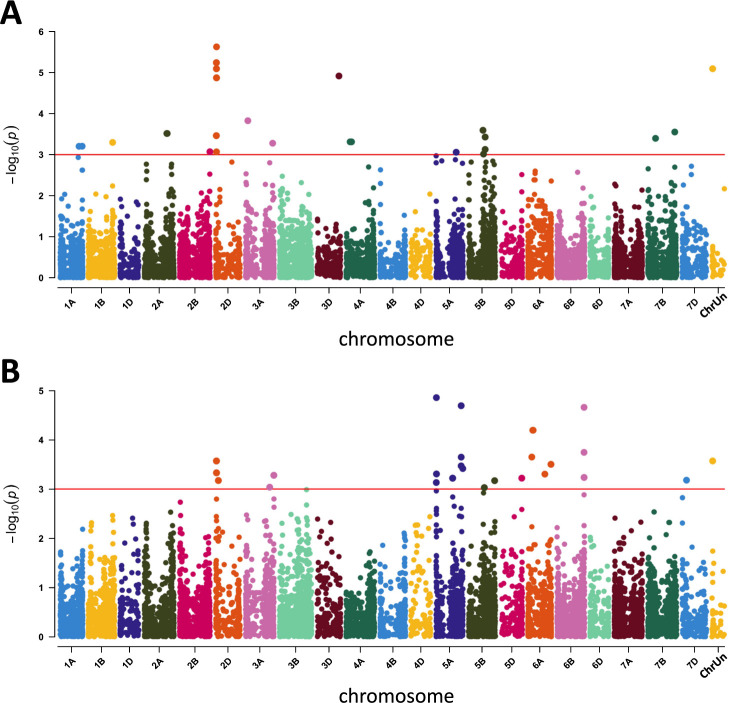
Manhattan plots of marker–trait associations. Association with spike length (SL) in 2018 (A). Association with culm length (CL) in 2018 (B). The horizontal red lines represent *p* values ≤0.001. ChrUn represents SNPs that map to sequences for which the attributed chromosome is unknown.

For CL and SL, we detected 72 and 67 MTAs distributed on 7 and 8 chromosomes (Fig. 4, Supplemental Tables 7, 8), 30 and 46 of which overlapped in a 2 Mbp region (22.1 to 24.0 Mbp) on the short arm of chromosome 2D. The amounts of phenotypic variance of CL and SL explained by these markers ranged from 13 to 25% (Supplemental Tables 7, 8). The remaining 21 MTAs for SL were distributed on the other 7 chromosomes, which explained 12 to 24% of the total phenotypic variance (Fig. 4A, Supplemental Table 8). We detected 42 MTAs for CL on six chromosomes other than 2D (Fig. 4B, Supplemental Table 7). Sixteen MTAs were located on the long arm of chromosome 5A (440–442 Mbp region), and several MTAs were detected on the long arms of 5B and 5D (Fig. 4B, Supplemental Table 7).

### Genotypic effects on spike length and culm length

As noted above, we found an overlap of the regions where multiple MTAs were stacked for SL and CL on chromosome 2D (Fig. 4, Supplemental Tables 7, 8). Recently, an *Rht8* gene was cloned and found to be located at approximately 25.6 Mbp on the chromosome 2DS distal end (Chai *et al.* 2022, Xiong *et al.* 2022), immediately adjacent to the overlapping MTA region for SL and CL from 22.1 to 24.0 Mbp. *Rht8* has pleiotropic effects on CL and SL (Kowalski *et al.* 2016, Wu *et al.* 2014, Zhai *et al.* 2016). On the other hand, the semidwarfness of Japanese wheat accessions is well known, and the JWC includes Norin 10, a cultivar famous for its role in the Green Revolution (Hedden 2003). However, we did not detect any MTAs on the chromosomes of homoeologous group 4, on which the semidwarf genes associated with the Green Revolution, *Rht-B1* and *Rht-D1*, were located. Therefore, we examined the relationships between *Rht8*, *Rht-D1*, and *Rht-B1* and phenotypic effects on SL and CL in the JWC population using the genes themselves or adjacent markers.

**Fig. 5.**
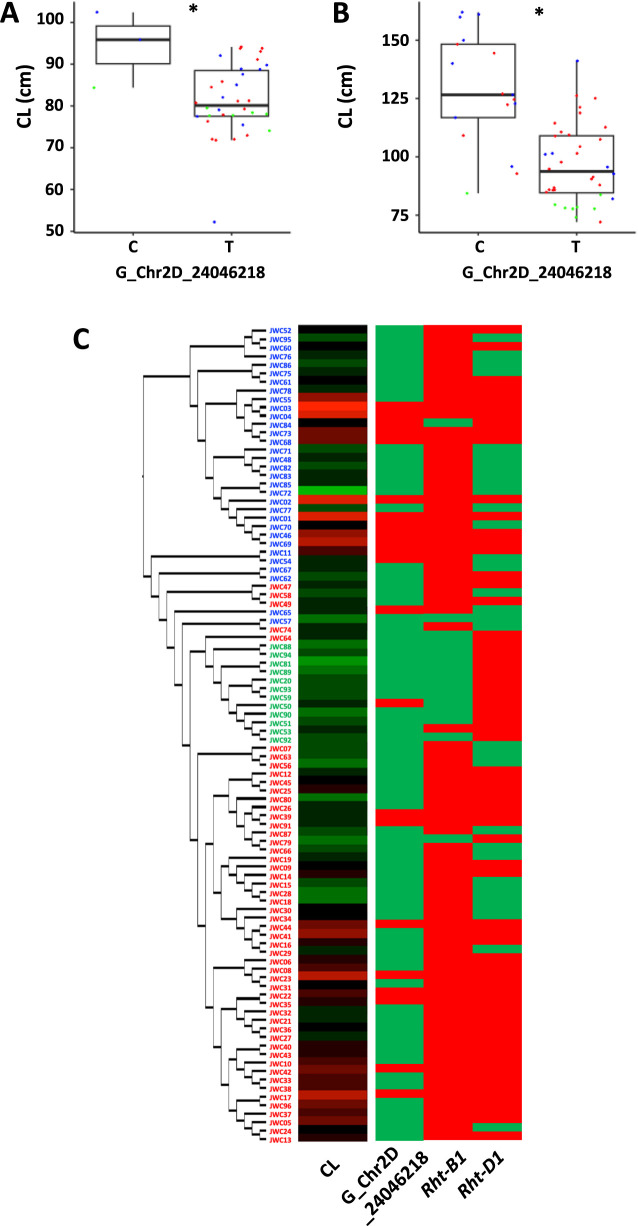
Relationship between culm length (CL) and genotypes of *Rht* genes. Comparison of CL between the genotype of G_Chr2D_24046218 in the accessions with *Rht-B1b* and *Rht-D1b* (A) and without *Rht-B1b* or *Rht-D1b* (B). Blue, green, and red dots indicate the accessions belonging to populations I, II, and III, respectively. Asterisks indicate a significant difference (*p* < 0.05). Heatmap of the CL in the JWC accessions (C). The columns of the heatmap are ordered based on the maximum likelihood phylogenetic tree, which is shown on the left with its branch lengths transformed to be proportional. Green and red genotypes indicate dwarf and wild-type alleles of each *Rht* gene, respectively. A heatmap has been derived from Fig. 3.

For *Rht8*, we detected the marker G_Chr2D_24046218, a common MTA for CL and SL closest to the *Rht8* position. This marker was associated with substantial differences in CL (Fig. 5A, 5B). There were significant differences in CL between the genotype of this marker regardless of the genotype of the *Rht-B1* or *Rht-D1* locus. The dwarfism genotype was distributed at a high frequency among the JWC accessions, 77.1% of G_Chr2D_24046218 (Fig. 5C).

The accessions with *Rht-B1b* or *Rht-D1b*, semidwarfism alleles, showed significantly reduced CL values compared to those of accessions without *Rht-B1b* and *Rht-D1b* (Supplemental Fig. 2A). The allele frequencies of *Rht-B1* and *Rht-D1* varied between population II and the other two populations (Supplemental Fig. 2B). Most accessions in population II had *Rht-B1b*, whereas there were no accessions with *Rht-D1b*. There was a large amount of variation in CL among accessions with wild-type alleles (*Rht-B1a*/*Rht-D1a*) and a high correlation between CL and DH (*r* = 0.88) (Supplemental Fig. 2C), which explains the variation in CL in accessions without semidwarfism alleles.

### Pedigree analysis of JWC accessions

We analyzed how each population was formed during the breeding process by combining the structural analysis results with lineage information. The accessions in population I mainly originated from Hokkaido, Tohoku, and parts of Kanto/Tosan such as Gunma, Niigata, and Nagano (Supplemental Table 1), where the climate is relatively cool throughout the year and winters can be relatively cold. We previously discussed in Kobayashi *et al.* (2016) that foreign accessions from Europe and North America were introduced into the breeding program to improve cold tolerance and snow mold resistance (Fukunaga and Inagaki 1985, Hoshino and Seko 1996). The four classical cultivars in Hokkaido, ‘Akagawa aka’ (JWC01), ‘Shirohada’ (JWC02), ‘Dawson 1’ (JWC03), and ‘Sapporo Harukomugi’ (JWC04), were selected as superior materials from introduced resources from the USA (Amano 2000). Therefore, these cultivars can be considered equivalent to foreign accessions. Most other accessions have foreign cultivars and above four accessions as parents or grandparents on their lineage (Supplemental Fig. 3), the genetic structure composition of which was substantially affected by the genetic background of the foreign accessions used for breeding.

Most classical varieties, excluding those from Hokkaido and modern breeders lines from Kanto to Kyushu belong to populations II and III (Supplemental Table 1). ‘Shin Chunaga’ (JWC26) and ‘Eshima Shinriki’ (JWC35) are landraces widely used as breeding material in the early days of modern wheat breeding in Japan (Supplemental Fig. 4). JWC26 largely contributed to the genetic background of many Japanese wheat cultivars, which has been supported by graphical genotyping analysis (Matsunaka *et al.* 2010). Around the same time, classical varieties were crossed with foreign cultivars, including ‘Velvet’ and ‘Australia 13’ (Supplemental Fig. 4). The pedigree indicated that the accessions in populations II and III are mainly composed of classical varieties and their offspring. Still, there is also an occasional contribution of foreign cultivars, which are involved in the early days of breeding.

**Fig. 6.**
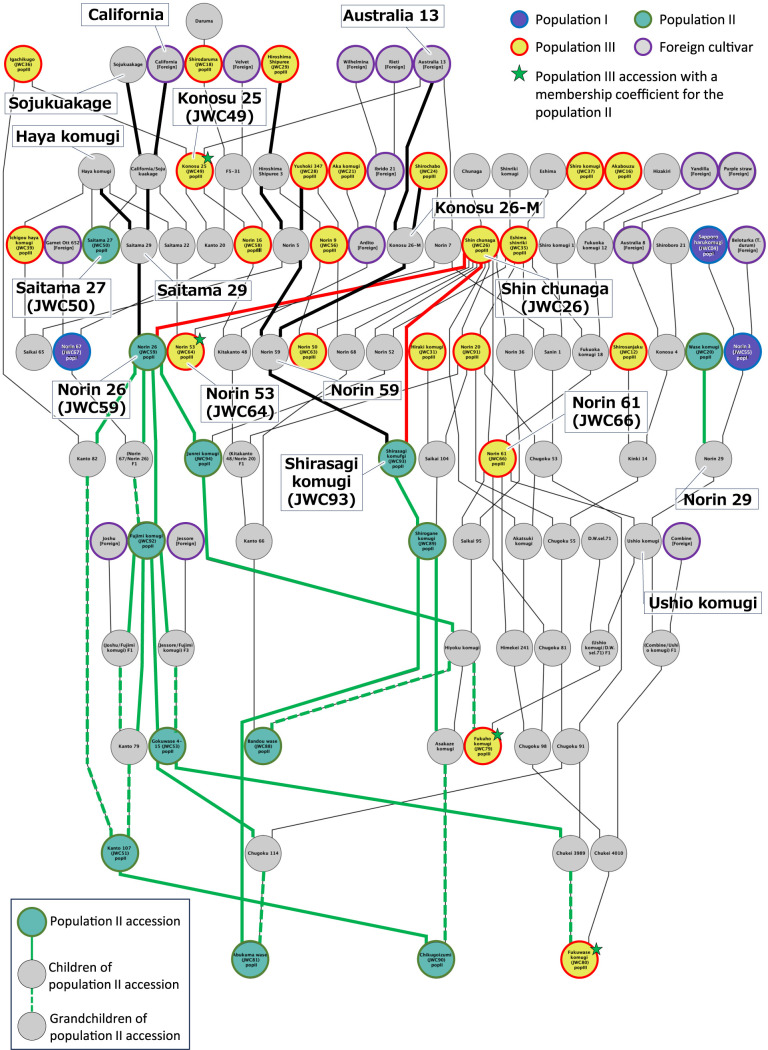
Pedigree of accessions belonging to population II and their related accessions. Green line (solid and dashed lines), linage of accessions in population II; red line, contribution of ‘Shin chunaga’ (JWC26) to ‘Norin 26’ (JWC59) and ‘Shirasagi komugi’ (JWC93); solid black line, linage of ‘Norin 26’ and ‘Shirasagi komugi’.

Although population II was closely related to population III in the pedigree, the population structure analysis separated population II from population III (Fig. 1). The accessions in population II displayed a characteristic phenotype compared to the others, and their early heading is remarkable (Fig. 2B). The pedigree showed that most accessions in population II have ‘Norin 26’ (JWC59) or ‘Shirasagi komugi’ (JWC93) as an ancestral crossing parent in their lineages (Fig. 6). JWC59 and JWC93 shared a common maternal line, ‘Shin Chunaga’ (JWC26). However, JWC26 belonged to population III (Fig. 1B), and its earliness was classified as medium rather than extreme. A paternal parent of JWC59 and JWC93 is ‘Saitama 29’ and ‘Norin 59’, respectively (Fig. 6). The pedigree of ‘Saitama 29’ is ‘California’/‘Sojukuakage’//‘Hayakomugi’. ‘Sojukuakage’ and ‘Hayakomugi’ are known as early-maturity landraces. The lineage of ‘Norin 59’ included ‘Australia 13’ in addition to classical accessions of population III (Fig. 6). This information revealed a strong influence of domestic accessions on the genetic background of JWC59 and JWC93 and the contribution of two foreign varieties, ‘California’ and ‘Australia13’. This combined genetic background might have shaped the characteristics of population II.

## Discussion

Our previous analysis of the population structure revealed that the JWC accessions could be divided into three groups: population I, the Hokkaido/Tohoku cultivar group; population III, landraces from southwestern Japan; and population II, the modern cultivar group, which shows an admixture pattern and is separated into subgroups IIa and IIb (Kobayashi *et al.* 2016). The results of the structural analyses in this study support this model, which showed that for populations I and III, each population consisted of a compact core group and admixed accessions. In contrast, population II was separated from the other populations into a small group (Fig. 1). All 12 accessions in population II were classified into previous population IIb, which could be characterized as a group of early-maturity varieties. The previous population structure at *K* = 7 indicated that population IIb exhibited a unique genetic structure (Kobayashi *et al.* 2016), which agrees with the results of this study (Fig. 1C). Pedigree analysis of population II accessions in this study suggested that two foreign cultivars, ‘California’ and ‘Australia 13’, contributed to the formation of the genetic structure of population II (Fig. 6). To characterize further the contribution of ‘California’ and ‘Australia 13’ to population II, we focused on three accessions, ‘Saitama 27’ (JWC50), ‘Konosu 25’ (JWC49) and ‘Norin 53’ (JWC64), although they are not directly related to the lineage of ‘Norin 26’ (JWC59) or ‘Shirasagi komugi’ (JWC93). JWC50 is classified into population II, and its pedigree is ‘California’/‘Sojukuakage’//‘Hayakomugi’. JWC49 is an offspring of ‘Australia 13’ (synonym: ‘Florence’) and had a high membership coefficient for population II (0.34), even though it was classified as a member of population III (Fig. 1B). JWC64, carrying a high membership coefficient for population II (0.32), has both ‘California’ and ‘Australia 13’ in its pedigree. Furthermore, a previous genetic study revealed that the allele of *Vrn-A1* conferring a spring growth habit was one of the genetic elements introduced from foreign cultivars such as ‘California’ and ‘Australia 13’ into traditional Japanese cultivars (Gotoh 1979). These findings support our hypothesis that the genetic structure composition of population II was substantially affected by ‘California’ and ‘Australia 13’, which produces a group of cultivars with distinctive early-maturity features.

Wheat pangenome studies have revealed genetic diversity among wheat accessions bred as part of global breeding programs. These studies revealed that the genetic structure of East Asian accessions such as ‘Norin 61’ (JWC66, population III) and ‘Chinese Spring’ (CS, JWC96, population III) are appreciably different from those of accessions from Europe, North America, and Australia (Balfourier *et al.* 2019, Walkowiak *et al.* 2020). Therefore, the extent of the contribution of foreign cultivars to breeding programs predominantly affects the population structure and genetic diversity of Japanese wheat cultivars. The contribution of foreign cultivars was more significant in population I than in populations II and III. The influence of foreign cultivars can also be seen in the breeding lineages, leading to modern cultivars belonging to populations II and III; however, these are distantly related (Fig. 6, Supplemental Figs. 3, 4). We previously observed that specific chromosomes and chromosome arms have lower SNP densities than others (Kobayashi *et al.* 2016). This result suggests that the chromosomes or regions needed for adaptation to the Japanese climate are conserved among all accessions, with limited variation. In contrast, the chromosomal segments controlling various requirements for modern cultivar breeding, such as flour quality to adapt to dietary changes in Japan and resistance to newly emerging diseases and pests, may vary significantly as a result of modern breeding. These considerations reflect the history of Japanese wheat breeding, in which modern Japanese cultivars have been developed based on the landraces belonging to population III and valuable genes have been incorporated from the foreign cultivars directly or via the exotic accessions in population I. The breeding of ‘Ushio komugi’ is a typical example, which is derived from a cross between JWC66 and ‘Norin 29’. JWC66 was a representative wheat cultivar in southwestern Japan, while ‘Norin 29’ was used to provide it with resistance to powdery mildew and leaf rust (Gotoh *et al.* 1968). Lineages of ‘Norin 29’ include two Population I accessions, ‘Norin 3’ (JWC55) and ‘Sapporo Harukomugi’ (JWC04) (Fig. 6, Supplemental Fig. 4). These three cultivars have a powdery mildew resistance gene *Pm3a* (Peusha *et al.* 1996), which is presumed to have been transmitted to ‘Ushio komugi’. Although the current evidence is insufficient to support the above considerations, expanding the genome-wide SNP data obtained in this study will allow us to identify more definitively essential and variable genomic regions in Japanese wheat cultivars in future studies.

The GWAS identified 220 MTAs for all examined traits, except SNS, with *p* values ≤0.001 (Supplemental Tables 6–12). However, no MTAs for any traits were statistically significant after Bonferroni multiple test correction. This is an unexpected result given the number of MTAs with *p* values ≤0.001. Only MTAs with weak effects may indicate that dominant alleles with strong effects on these traits do not differentiate the Japanese wheat accessions but that rare minor alleles with weak effects in a relatively homogeneous genetic background do. Furthermore, these results might be due to population size, allele frequency, and population structure individually or in combination. The most interesting finding from GWAS was the overlapping of multiple MTAs for SL and CL in chromosome 2D (Fig. 4, Supplemental Tables 7, 8), in which *Rht8* was located.

*Rht8*, recently cloned (Chai *et al.* 2022, Xiong *et al.* 2022), was derived from the Japanese cultivar ‘Akakomugi’ (JWC21) and introduced into southern European wheat breeding in the 1930s, contributing to the development of semidwarf cultivars (Borojevic and Borojevic 2005). Other semidwarfism genes, such as *Rht1* (*Rht-B1*) and *Rht2* (*Rht-D1*) on chromosomes 4B and 4D, also known for their contribution to the Green Revolution, also originated from the Japanese cultivar ‘Norin 10’ (JWC57) (Hedden 2003). These three semidwarfism genes play significant roles in wheat breeding worldwide. Although they are all known to have originated in Japanese wheat accessions, it remains unclear how they evolved within the Japanese wheat lineage. In this study, we found MTAs for CL on chromosome 2D but not on 4B and 4D; therefore, we investigated the relationship between CL phenotypes and genotypes of the three *Rht* genes, *Rht-B1*, *Rht-D1*, and *Rht8*, in the JWC (Fig. 5, Supplemental Table 1). *Rht-B1* and *Rht-D1* show unique distribution patterns for each gene. The semidwarfism *Rht-D1b* allele was absent in population II, while the semidwarfism *Rht-B1b* allele was clustered in population II. The accessions belonging to population II accounted for only 1/8 of the JWC accessions. We assume that these biases in population size, allele frequencies, and population structure are why MTA was not detected in 4B and 4D. In contrast, the *Rht8* semidwarf genotype was widely distributed and more abundant in population III, which consisted of original Japanese landraces. These results indicate that *Rht-D1b* and *Rht-B1b* can be used selectively in the breeding process to shorten culms further. The breeding use of *Rht-B1b* may be more recent than that of *Rht-D1b*. From the combined data for the three *Rht* genes in the JWC accessions, we can explain the Japanese breeding process to shorten the CL as follows: *Rht8*, which has a milder effect on the CL than *Rht1*, was widely distributed before modern Japanese wheat breeding because the dwarfing caused by *Rht1* is too strong and has a negative effect on yield performance, and *Rht8* plays a more significant role than *Rht-D1* and *Rht-B1* before the fertilizer-intensive period. Later, when a shorter culm was needed for more fertilizer use, breeders may have controlled the CL by adding *Rht-D1b* or *Rht-B1b* to *Rht8*.

The GWAS for CL revealed multiple MTAs on the long arm of homoeologous chromosome group 5 (Supplemental Table 5), the position of which does not coincide with those of the known *Rht* genes often used for wheat breeding, such as *Rht-B1*, *Rht-D1*, and *Rht8*. To date, 25 dwarfism genes have been identified in wheat (McIntosh *et al.* 2013, Mo *et al.* 2018), and three *Rht* genes were identified in the homoeologous chromosome group 5, *Rht9* and *Rht12* on 5AL, and *Rht23* on 5DL. *Rht23* was located in the 533 Mbp region of 5DL (Chen *et al.* 2015), which is consistent with the MTA of CL on 5DL (Supplemental Table 7). On the other hand, *Rht23* has been postulated to be a *Q* homoeolog that shows pleiotropic effects on plant height and spike compactness (Zhao *et al.* 2018), but in the JWC, no allelic effect has been found on 5DL, leading to a change in spike morphology, suggesting that the CL MTA on 5DL found in this study is different from *Rht23*.

*Rht9* was mapped to chromosome arm 5AL in the Chuan Mai 18 (*Rht8*) × Mara (*Rht8/Rht9*) population and linked to marker BARC151, which is located in the 560 Mbp region of 5AL (Ellis *et al.* 2005), while *Rht12* was closely linked with the Xw5ac207 SSR marker located in the 700 Mbp region at the terminal end of chromosome arm 5AL (Sun *et al.* 2019). Interestingly, both genes are GA-sensitive genes, although both were slightly distant from the region (440–442 Mbp) where a large number of CL MTAs have been detected. The detection of MTAs, possibly corresponding to *Rht9*, is particularly noteworthy. *Rht9*, together with *Rht8*, has made a significant contribution to semidwarf wheat breeding in southern Europe, including Italy and Yugoslavia, which originated from the Japanese wheat cultivar ‘Akakomugi’ (Worland 1986). It is unknown how widespread it is worldwide, but it is an interesting target for future research as a semidwarfism gene originating in Japan and contributing greatly to wheat breeding.

By combining available high-throughput genotyping data (Kobayashi *et al.* 2016 and in this study) with the latest genomic information (IWGSC 2018, Zhu *et al.* 2021), this study revealed the genomic diversity of the JWC in more detail. Genomic analyses, including GWAS, have revealed the detailed population structure of Japanese wheat accessions and outlined the Japanese wheat breeding process from a genomic viewpoint. The original Japanese landraces were adapted to a climate with moderate winters and humid springs/summers, which are prevalent in southwestern Japan. Few landraces and classical cultivars have been repeatedly used to breed modern cultivars in this area. On the other hand, superior foreign cultivars were introduced and used as breeding materials in northeastern Japan at the beginning of the breeding history. However, the subsequent breeding of most modern cultivars has been based on crosses between indigenous and foreign cultivars to introduce specific traits, such as disease resistance. Variations in *Rht8*, *Rht-B1*, and *Rht-D1* show how these three semidwarfism genes have been utilized differently in Japanese wheat breeding. These results suggest that useful genetic resources that have not been utilized worldwide may exist in the Japanese lineage. Thus, the JWC collection represents a valuable germplasm set for understanding the genetic diversity within Japanese wheat accessions and developing new wheat cultivars. On the other hand, biases due to the small population size and distinctive population structure revealed by GWAS represent a weakness in utilizing the JWC in a wide range of applications. This can be overcome by expanding the population size while considering population structure. In addition, the JWC does not include recently bred cultivars, as more than fifteen years have passed since the selection of the accessions. Therefore, updating the composition of the collection should be considered. Furthermore, the high-resolution genome sequence of ‘Norin 61’ (JWC66) (Shimizu *et al.* 2021) was recently decoded. Combining this genomic information with the JWC will significantly contribute to future breeding research by increasing our knowledge of genetic diversity within Japanese wheat accessions.

## Author Contribution Statement

HH and FK conceived of the study. NM and TM generated and analyzed the bioinformatics data. FK and HH conducted phenotypic surveys in the field. HH, NM, and FK drafted the manuscript.

## Supplementary Material

Supplemental Figures

Supplemental Tables

## Figures and Tables

**Table 1.** Descriptive sttistics of eight traits of the JWC accessions in five years

Traits*^a^*	Year	Range			Mean	SD*^b^*	CV (%)*^c^*	*h*^2^ (%)*^d^*	Significance*^e^*	
									Genotype	Year
DH (days)	2016	163.0	–	194.7	177.4	7.89	4.5	90.4	**	**
	2017	163.0	–	195.3	179.6	6.90	3.8			
	2018	152.5	–	186.0	165.1	6.35	3.8			
	2019	153.7	–	197.3	179.6	8.29	4.6			
	2020	145.7	–	190.0	169.8	10.40	6.1			
CL (cm)	2016	61.0	–	177.3	111.8	26.84	24.0	90.0	**	**
	2017	34.3	–	132.5	78.8	19.17	24.3			
	2018	49.6	–	145.2	86.3	21.04	24.4			
	2019	61.2	–	187.9	110.5	29.90	27.1			
	2020	54.9	–	181.2	102.7	27.80	27.1			
SL (cm)	2016	7.0	–	15.0	10.6	1.72	16.2	86.9	**	**
	2017	7.0	–	16.4	12.1	1.99	16.4			
	2018	7.3	–	16.4	10.6	2.24	21.1			
	2019	7.8	–	18.2	11.9	1.94	16.3			
	2020	7.5	–	17.2	11.8	2.16	18.3			
SNS (spikelets/spike)	2016	14.7	–	26.7	22.1	2.72	12.3	72.8	**	**
	2017	16.0	–	27.7	22.7	2.31	10.2			
	2018	16.5	–	26.0	21.3	1.76	8.3			
	2019	18.7	–	29.0	22.9	2.19	9.6			
	2020	17.0	–	28.3	22.3	2.42	10.9			
SD (spikes/cm)	2016	1.51	–	3.37	2.13	0.39	18.3	86.9	**	**
	2017	1.37	–	3.25	1.93	0.39	20.0			
	2018	1.23	–	3.01	2.08	0.39	18.5			
	2019	1.38	–	3.30	1.97	0.35	18.0			
	2020	1.27	–	2.97	1.94	0.36	18.3			
GNS (grains/spike)	2016	34.6	–	98.1	62.4	11.41	18.3	71.8	**	**
	2017	35.4	–	93.6	67.9	10.84	16.0			
	2018	37.2	–	100.8	67.6	11.38	16.8			
	2019	39.1	–	96.6	69.8	12.66	18.1			
	2020	28.5	–	99.0	69.1	13.60	19.7			
GNSL (grains/spikelet)	2016	1.45	–	3.74	2.85	0.51	17.8	71.3	**	**
	2017	1.50	–	4.49	3.02	0.56	18.6			
	2018	1.67	–	4.38	3.18	0.48	15.2			
	2019	1.52	–	4.11	3.06	0.55	17.9			
	2020	1.46	–	4.57	3.13	0.65	20.8			
TKW (g)	2016	24.8	–	54.1	38.3	6.65	17.4	79.2	**	**
	2017	25.7	–	54.5	38.6	6.05	15.7			
	2018	31.5	–	54.9	40.7	5.23	12.9			
	2019	25.6	–	47.5	36.1	4.75	13.2			
	2020	26.8	–	58.0	37.5	6.22	16.6			

*^a^* DH, days to heading; CL, culm length; SL, spike length; SNS, spikelet numbers per spike; SD, spike density; GNS, grain numbers per spike; GNSL, grain numbers per spikelet; TKW, thousand-kernel weight. *^b^* Standard deviation. *^c^* Coefficint of variation. *^d^* Broad-sense heritability. *^e^*Significance, ** at *p* < 0.01.

**Table 2.** Correlation coefficients of 5-year means for eight traits in the JWC accessions

	DH	CL	SL	SNS	SD	GNS	GNSL	TKW
DH	1							
CL	0.721***	1						
SL	0.452***	0.518***	1					
SNS	0.657***	0.501***	0.230*	1				
SD	0.008	–0.119	–0.811***	0.348***	1			
GNS	–0.286**	–0.294**	–0.416***	0.244*	0.481***	1		
GNSL	–0.663***	–0.563***	–0.531***	–0.355***	0.254*	0.816***	1	
TKW	0.198	0.317**	0.471***	–0.093	–0.475***	–0.521***	–0.431***	1

DH, days to heading; CL, culm length; SL, spike length; SNS, spikelet numbers per spike; SD, spike density; GNS, grain numbers per spike; GNSL, grain numbers per spikelet; TKW, thousand-kernel weight. *, ** and ***; significane at *p* < 0.05, 0.01, and 0.001, respectively.
