# Supplementary material for: Genomic diversity of the Japanese wheat core collection and selection of alleles for agronomic traits in the breeding process
Source: Breed Sci. 2024 Jun 25;74(3):259–73. doi: 10.1270/jsbbs.23064 (PMC11561409; doi:10.1270/jsbbs.23064)
Supplement: Supplementary file 1 — Supplemental Figures [file 74_259-s1.pdf]

Mizuno et al./Breeding Science
doi: 10.1270/jsbbs.23064

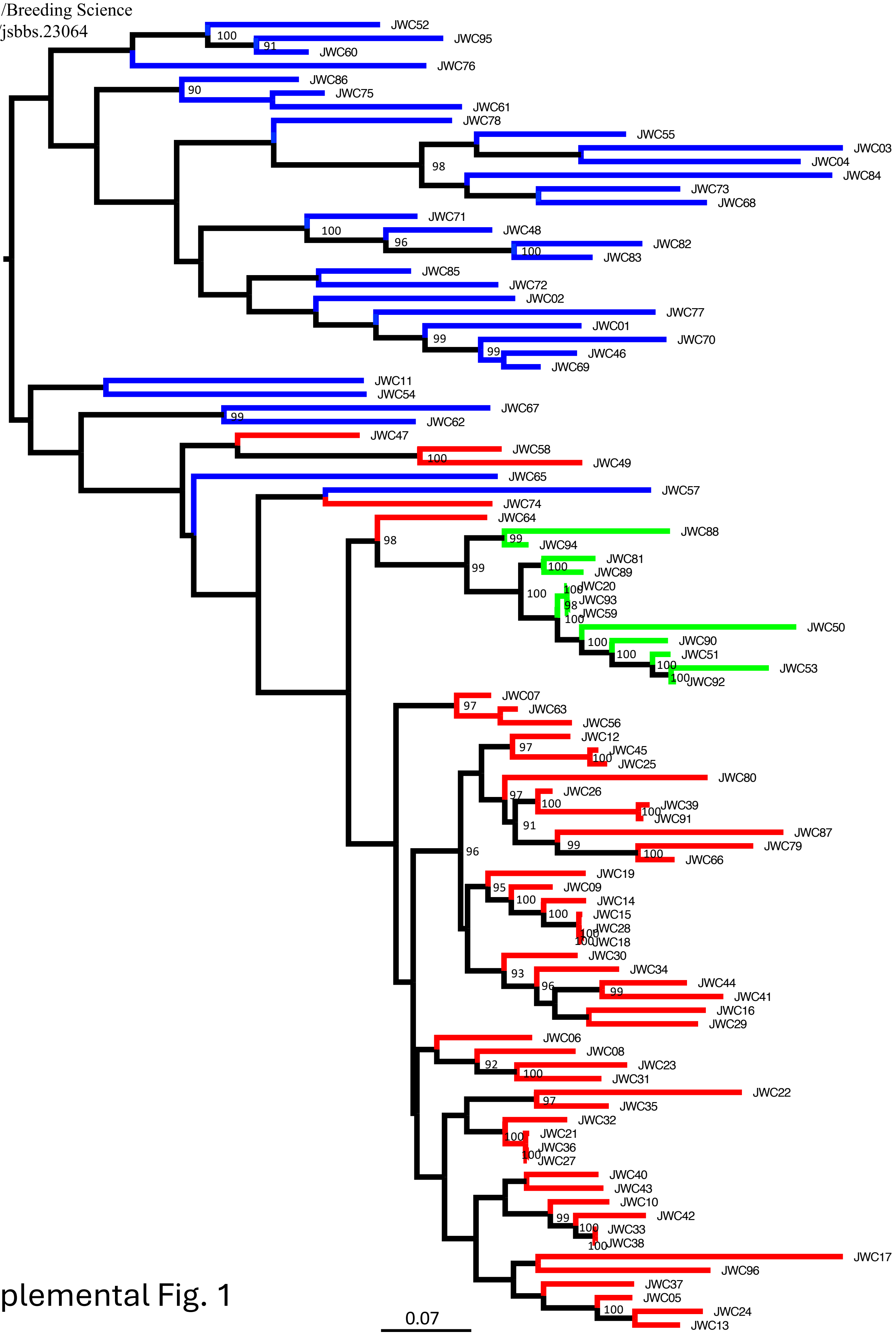

Supplemental Fig. 1

Supplemental Figure 1. Maximum likelihood tree of the 96 JWC accessions. Numbers above branches show bootstrap values based on 1000 replicates (not shown if < 90%). Blue, green, and red branches indicate the accessions belonging to populations I, II, and III, respectively.

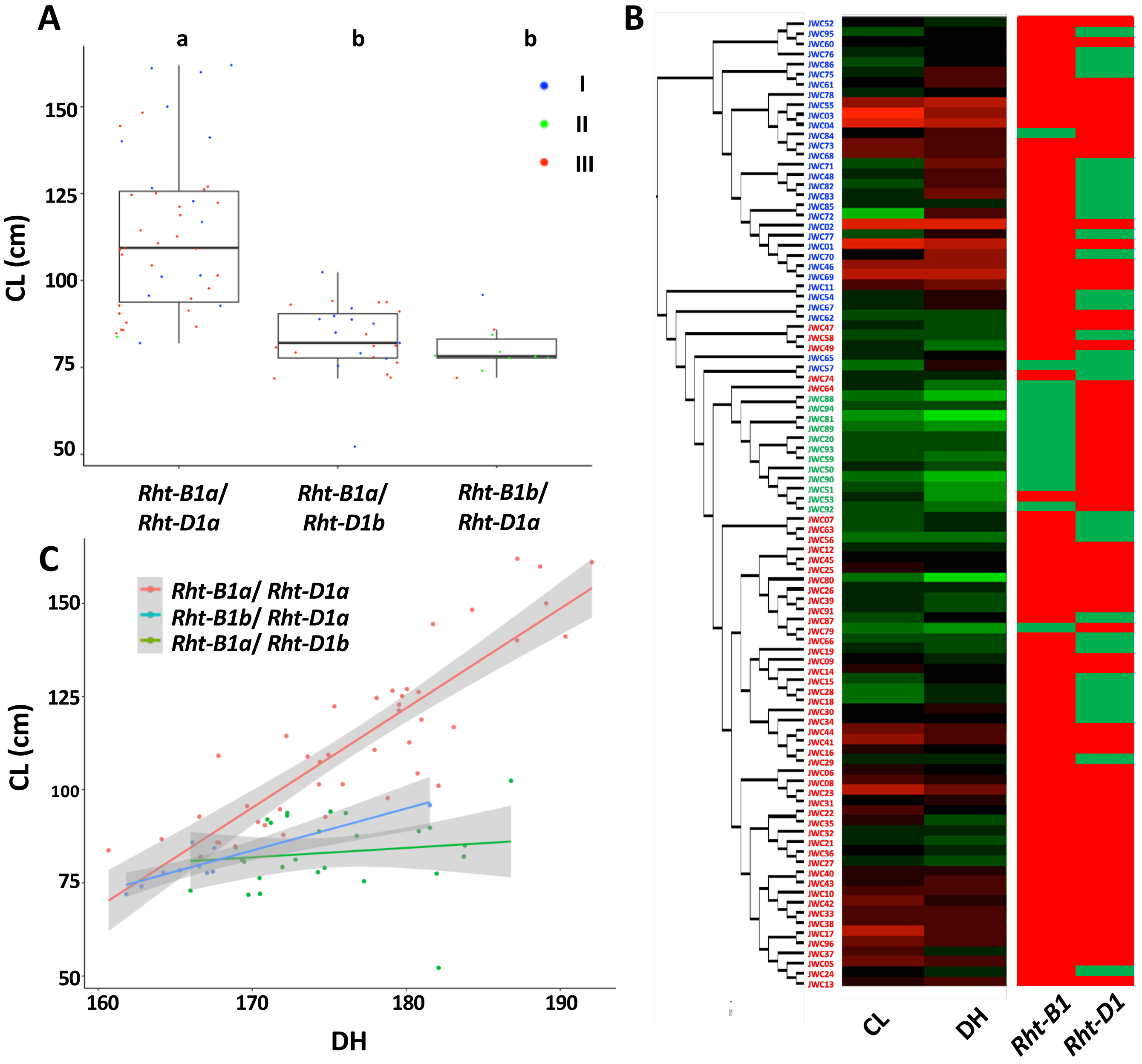

Supplemental Fig. 2

Supplemental Figure 2. Genotypes of *Rht-1* genes and days to heading (DH) significantly affect culm length (CL). Comparison of CL among the allele combination of *Rht-B1* and *Rht-D1*. Blue, green, and red dots indicate the accessions belonging to populations I, II, and III, respectively (A). Mean values with the same letters are not significantly different ($P > 0.05$) (Tukey–Kramer's HSD test). Heatmap of CL and DH in the JWC accessions (B). The columns of the heatmap are ordered based on the maximum likelihood tree, shown on the left, with its branch lengths transformed to be proportional. Missing genotypes of *Rht-B1* and *Rht-D1* are shown in gray. Red and green genotypes indicate dwarf and wild-type alleles of each *Rht-1* gene, respectively. Heatmap has been derived from Fig. 3. Relationship between CL and DH for allele combination of *Rht-B1* and *Rht-D1* (C). The gray shaded area represents the 95% confidence interval.

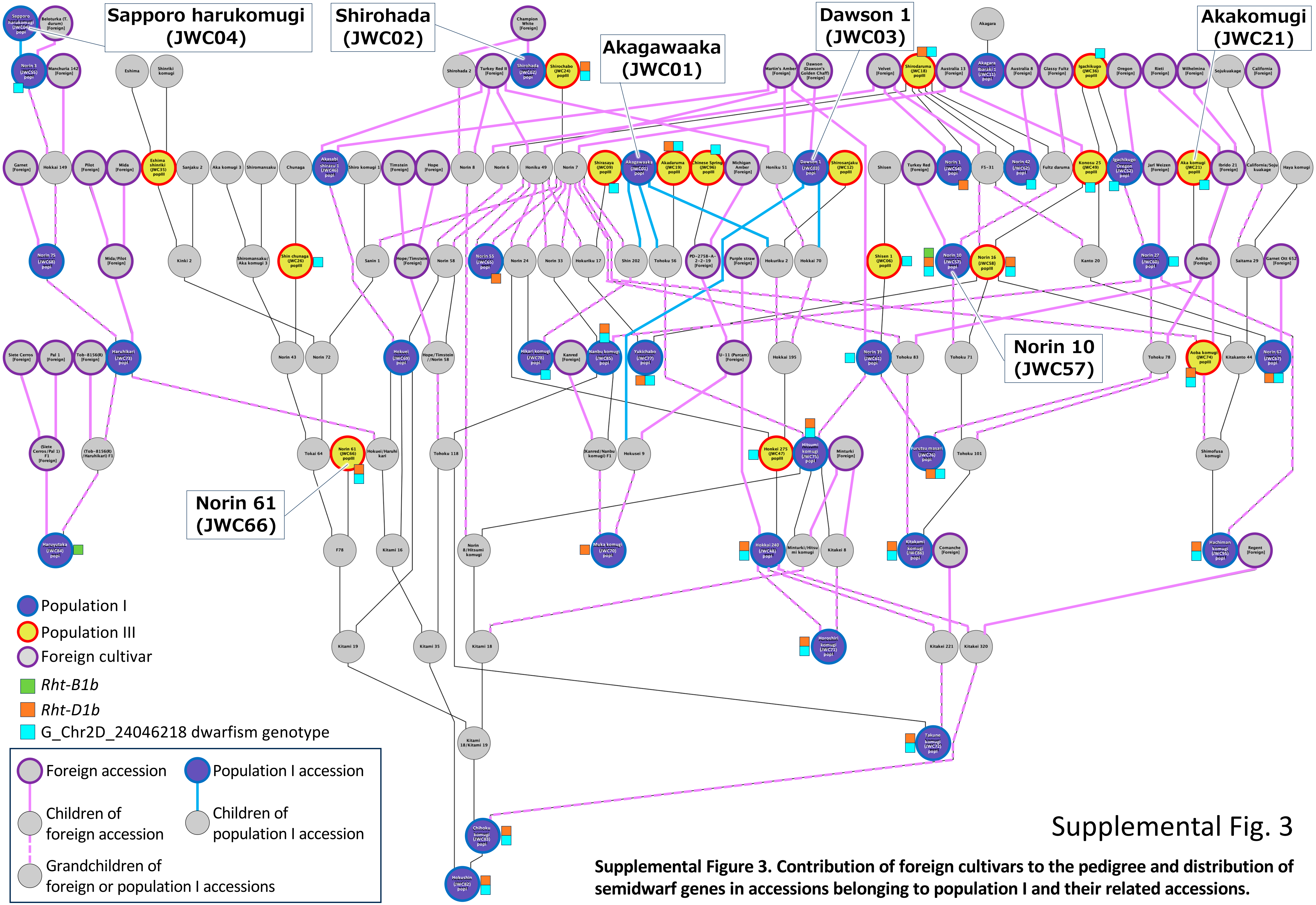

Supplemental Fig. 3

Supplemental Figure 3. Contribution of foreign cultivars to the pedigree and distribution of semidwarf genes in accessions belonging to population I and their related accessions.

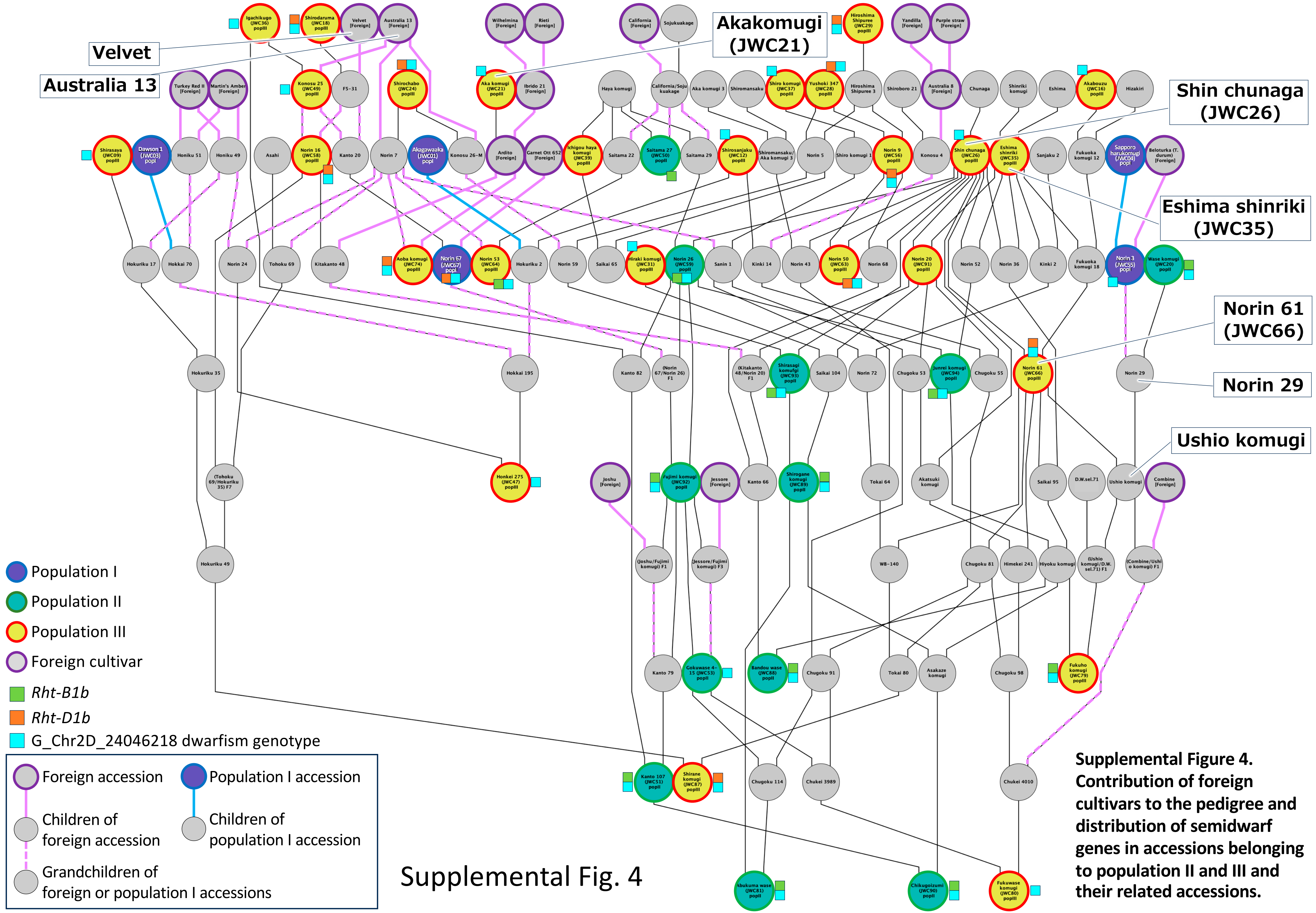

Supplemental Fig. 4

Supplemental Figure 4. Contribution of foreign cultivars to the pedigree and distribution of semidwarf genes in accessions belonging to population II and III and their related accessions.
