# Supplementary material for: Genomic diversity of the Japanese wheat core collection and selection of alleles for agronomic traits in the breeding process
Source: Breed Sci. 2024 Jun 25;74(3):259–73. doi: 10.1270/jsbbs.23064 (PMC11561409; doi:10.1270/jsbbs.23064)
Supplement: Supplementary file 2 — Supplemental Tables [file 74_259-s2.pdf]

doi: 10.1270/jsbbs.23064

Supplemental Table 1. Accessions in the NARO Japanese wheat core collection

Accession ID[1)]	Cultivar name	Status	Category[2)]	Locality	Area[3)]	Population[4)]	*Rht-D1* [5)]	*Rht-B1* [5)]
JWC 01	Akagawa aka	Landrace	Classical	Hokkaido	Hokkaido	I	*Rht-D1a*	*Rht-B1a*
JWC 02	Shirohada	Unknown	Classical	Hokkaido	Hokkaido	I	*Rht-D1a*	*Rht-B1a*
JWC 03	Dawson 1	Pure selected line	Classical	Hokkaido	Hokkaido	I	*Rht-D1a*	*Rht-B1a*
JWC 04	Sapporo Harukomugi	Breeders line	Classical	Hokkaido	Hokkaido	I	*Rht-D1a*	*Rht-B1a*
JWC 05	Soshu 2	Pure selected line	Classical	Aomori	Tohoku	III	*Rht-D1a*	*Rht-B1a*
JWC 06	Shisen 1	Pure selected line	Classical	Iwate	Tohoku	III	*Rht-D1a*	*Rht-B1a*
JWC 07	Zairai Fultz	Landrace	Classical	Iwate	Tohoku	III	*Rht-D1b*	*Rht-B1a*
JWC 08	Nishimura	Breeders line	Classical	Yamagata	Tohoku	III	*Rht-D1a*	*Rht-B1a*
JWC 09	Shirasaya	Unknown	Classical	Ibaraki	Kanto/Tosan	III	*Rht-D1a*	*Rht-B1a*
JWC 10	Fukoku	Breeders line	Classical	Ibaraki	Kanto/Tosan	III	*Rht-D1a*	*Rht-B1a*
JWC 11	Akagara Ibaraki 1	Pure selected line	Classical	Ibaraki	Kanto/Tosan	I	*Rht-D1a*	*Rht-B1a*
JWC 12	Shirosanjaku	Unknown	Classical	Ibaraki	Kanto/Tosan	III	*Rht-D1a*	*Rht-B1a*
JWC 13	Akaboro 1	Pure selected line	Classical	Tochigi	Kanto/Tosan	III	*Rht-D1a*	*Rht-B1a*
JWC 14	Nittawase	Landrace	Classical	Gunma	Kanto/Tosan	III	*Rht-D1a*	*Rht-B1a*
JWC 15	Sunekiri 15	Pure selected line	Classical	Gunma	Kanto/Tosan	III	*Rht-D1b*	*Rht-B1a*
JWC 16	Akabouzu	Breeders line	Classical	Saitama	Kanto/Tosan	III	*Rht-D1a*	*Rht-B1a*
JWC 17	Hosogara	Breeders line	Classical	Chiba	Kanto/Tosan	III	*Rht-D1a*	*Rht-B1a*
JWC 18	Shirodaruma	Breeders line	Classical	Kanagawa	Kanto/Tosan	III	*Rht-D1b*	*Rht-B1a*
JWC 19	Akadaruma	Breeders line	Classical	Kanagawa	Kanto/Tosan	III	*Rht-D1b*	*Rht-B1a*
JWC 20	Wase komugi	Breeders line	Classical	Kanagawa	Kanto/Tosan	II	*Rht-D1a*	*Rht-B1b*
JWC 21	Aka komugi	Landrace	Classical	Yamanashi	Kanto/Tosan	III	*Rht-D1a*	*Rht-B1a*
JWC 22	Shibushirazu	Breeders line	Classical	Nagano	Kanto/Tosan	III	*Rht-D1a*	*Rht-B1a*
JWC 23	Koshigun zairaishu	Landrace	Classical	Niigata	Kanto/Tosan	III	*Rht-D1a*	*Rht-B1a*
JWC 24	Shirochabo	Unknown	Classical	Mie	Tokai/Hokuriku	III	*Rht-D1b*	*Rht-B1a*
JWC 25	Sakobore	Breeders line	Classical	Shizuoka	Tokai/Hokuriku	III	*Rht-D1a*	*Rht-B1a*
JWC 26	Shin Chunaga	Pure selected line	Classical	Hyogo	Kinki/Chugoku/Shikoku	III	*Rht-D1a*	*Rht-B1a*
JWC 27	Hatakeda komugi	Breeders line	Classical	Okayama	Kinki/Chugoku/Shikoku	III	*Rht-D1a*	*Rht-B1a*
JWC 28	Yushoki 347	Unknown	Classical	Okayama	Kinki/Chugoku/Shikoku	III	*Rht-D1b*	*Rht-B1a*
JWC 29	Hiroshima Shipree	Unknown	Classical	Hiroshima	Kinki/Chugoku/Shikoku	III	*Rht-D1b*	*Rht-B1a*
JWC 30	Mubouchinko	Breeders line	Classical	Hiroshima	Kinki/Chugoku/Shikoku	III	*Rht-D1b*	*Rht-B1a*
JWC 31	Hiraki komugi	Landrace	Classical	Tottori	Kinki/Chugoku/Shikoku	III	*Rht-D1a*	*Rht-B1a*
JWC 32	Nakasoshu	Unknown	Classical	Kagawa	Kinki/Chugoku/Shikoku	III	*Rht-D1a*	*Rht-B1a*
JWC 33	Homan	Unknown	Classical	Tochigi	Kanto/Tosan	III	*Rht-D1a*	*Rht-B1a*
JWC 34	Sekichiku 1	Pure selected line	Classical	Aichi	Tokai/Hokuriku	III	*Rht-D1b*	*Rht-B1a*
JWC 35	Eshima shinriki	Breeders line	Classical	Fukuoka	Kyushu	III	*Rht-D1a*	*Rht-B1a*
JWC 36	Igachikugo	Unknown	Classical	Saga	Kyushu	III	*Rht-D1a*	*Rht-B1a*
JWC 37	Shiro komugi	Landrace	Classical	Saga	Kyushu	III	*Rht-D1a*	*Rht-B1a*
JWC 38	Chikuzen	Landrace	Classical	Saga	Kyushu	III	*Rht-D1a*	*Rht-B1a*
JWC 39	Ichigo Haya komugi	Pure selected line	Classical	Kumamoto	Kyushu	III	*Rht-D1a*	*Rht-B1a*
JWC 40	Shirobunbu	Breeders line	Classical	Kumamoto	Kyushu	III	*Rht-D1a*	*Rht-B1a*
JWC 41	Aso zairai (yuubou kappu)	Landrace	Classical	Kumamoto	Kyushu	III	*Rht-D1a*	*Rht-B1a*
JWC 42	Sotome	Breeders line	Classical	Nagasaki	Kyushu	III	*Rht-D1a*	*Rht-B1a*
JWC 43	Sadabozu	Landrace	Classical	Miyazaki	Kyushu	III	*Rht-D1a*	*Rht-B1a*
JWC 44	Nobeokabouzu komugi	Landrace	Classical	Miyazaki	Kyushu	III	*Rht-D1a*	*Rht-B1a*
JWC 45	Sakigake 1	Pure selected line	Classical	Kagoshima	Kyushu	III	*Rht-D1a*	*Rht-B1a*
JWC 46	Akasabishirazu 1	Breeders line	Modern	Hokkaido	Hokkaido	I	*Rht-D1a*	*Rht-B1a*
JWC 47	Honkei 275	Breeders line	Modern	Hokkaido	Hokkaido	III	*Rht-D1a*	*Rht-B1a*
JWC 48	Hokkai 240	Breeders line	Modern	Hokkaido	Hokkaido	I	*Rht-D1b*	*Rht-B1a*
JWC 49	Konosu 25	Breeders line	Modern	Saitama	Kanto/Tosan	III	*Rht-D1a*	*Rht-B1a*
JWC 50	Saitama 27	Breeders line	Modern	Saitama	Kanto/Tosan	II	*Rht-D1a*	*Rht-B1b*
JWC 51	Kanto 107	Breeders line	Modern	Ibaraki	Kanto/Tosan	II	*Rht-D1a*	*Rht-B1b*
JWC 52	Igachikugo Oregon	Breeders line	Modern	Nagano	Kanto/Tosan	I	*Rht-D1a*	*Rht-B1a*
JWC 53	Gokuwase 4-15	Breeders line	Modern	Hiroshima	Kinki/Chugoku/Shikoku	II	*Rht-D1a*	*Rht-B1a*
JWC 54	Komugi Norin 1	Breeders line	Modern	Iwate	Tohoku	I	*Rht-D1b*	*Rht-B1a*
JWC 55	Harumakikomugi Norin 3	Breeders line	Modern	Hokkaido	Hokkaido	I	*Rht-D1a*	*Rht-B1a*
JWC 56	Komugi Norin 9	Breeders line	Modern	Aichi	Tokai/Hokuriku	III	*Rht-D1b*	*Rht-B1a*
JWC 57	Komugi Norin 10	Breeders line	Modern	Iwate	Tohoku	I	*Rht-D1b*	*Rht-B1b*

JWC 58	Komugi Norin 16	Breeders line	Modern	Gunma	Kanto/Tosan	III	*Rht-D1b*	*Rht-B1a*
JWC 59	Komugi Norin 26	Breeders line	Modern	Nara	Kinki/Chugoku/Shikoku	II	*Rht-D1a*	*Rht-B1b*
JWC 60	Komugi Norin 27	Breeders line	Modern	Iwate	Tohoku	I	*Rht-D1a*	*Rht-B1a*
JWC 61	Komugi Norin 39	Breeders line	Modern	Iwate	Tohoku	I	*Rht-D1a*	*Rht-B1a*
JWC 62	Koshitsukomugi Norin 42	Breeders line	Modern	Gunma	Kanto/Tosan	I	*Rht-D1a*	*Rht-B1a*
JWC 63	Komugi Norin 50	Breeders line	Modern	Gunma	Kanto/Tosan	III	*Rht-D1b*	*Rht-B1a*
JWC 64	Komugi Norin 53	Breeders line	Modern	Aichi	Tokai/Hokuriku	III	*Rht-D1a*	*Rht-B1b*
JWC 65	Komugi Norin 55	Breeders line	Modern	Iwate	Tohoku	I	*Rht-D1b*	*Rht-B1a*
JWC 66	Komugi Norin 61	Breeders line	Modern	Saga	Kyushu	III	*Rht-D1b*	*Rht-B1a*
JWC 67	Komugi Norin 67	Breeders line	Modern	Gunma	Kanto/Tosan	I	*Rht-D1b*	*Rht-B1a*
JWC 68	Harumakikomugi Norin 75	Breeders line	Modern	Hokkaido	Hokkaido	I	*Rht-D1a*	*Rht-B1a*
JWC 69	Hokuei	Breeders line	Modern	Hokkaido	Hokkaido	I	*Rht-D1a*	*Rht-B1a*
JWC 70	Muka komugi	Breeders line	Modern	Hokkaido	Hokkaido	I	*Rht-D1b*	*Rht-B1a*
JWC 71	Horoshiri komugi	Breeders line	Modern	Hokkaido	Hokkaido	I	*Rht-D1b*	*Rht-B1a*
JWC 72	Takune komugi	Breeders line	Modern	Hokkaido	Hokkaido	I	*Rht-D1b*	*Rht-B1a*
JWC 73	Haruhikari	Breeders line	Modern	Hokkaido	Hokkaido	I	*Rht-D1a*	*Rht-B1a*
JWC 74	Aoba komugi	Breeders line	Modern	Iwate	Tohoku	III	*Rht-D1b*	*Rht-B1a*
JWC 75	Hitsumi komugi	Breeders line	Modern	Iwate	Tohoku	I	*Rht-D1b*	*Rht-B1a*
JWC 76	Furutsu masari	Breeders line	Modern	Iwate	Tohoku	I	*Rht-D1b*	*Rht-B1a*
JWC 77	Yukichabo	Breeders line	Modern	Niigata	Kanto/Tosan	I	*Rht-D1b*	*Rht-B1a*
JWC 78	Hikari komugi	Breeders line	Modern	Niigata	Kanto/Tosan	I	*Rht-D1a*	*Rht-B1a*
JWC 79	Fukuho komugi	Breeders line	Modern	Saitama	Kanto/Tosan	III	*Rht-D1a*	*Rht-B1b*
JWC 80	Fukuwase komugi	Breeders line	Modern	Hiroshima	Kinki/Chugoku/Shikoku	III	*Rht-D1a*	*Rht-B1a*
JWC 81	Abukumawase	Breeders line	Modern	Fukuoka	Kyushu	II	*Rht-D1a*	*Rht-B1b*
JWC 82	Hokushin	Breeders line	Modern	Hokkaido	Hokkaido	I	*Rht-D1b*	*Rht-B1a*
JWC 83	Chihoku komugi	Breeders line	Modern	Hokkaido	Hokkaido	I	*Rht-D1b*	*Rht-B1a*
JWC 84	Haruyutaka	Breeders line	Modern	Hokkaido	Hokkaido	I	*Rht-D1a*	*Rht-B1b*
JWC 85	Nanbu komugi	Breeders line	Modern	Iwate	Tohoku	I	*Rht-D1b*	*Rht-B1a*
JWC 86	Kitakami komugi	Breeders line	Modern	Iwate	Tohoku	I	*Rht-D1b*	*Rht-B1a*
JWC 87	Shirane komugi	Breeders line	Modern	Nagano	Kanto/Tosan	III	*Rht-D1b*	*Rht-B1a*
JWC 88	Bando wase	Breeders line	Modern	Ibaraki	Kanto/Tosan	II	*Rht-D1a*	*Rht-B1b*
JWC 89	Shirogane komugi	Breeders line	Modern	Fukuoka	Kyushu	II	*Rht-D1a*	*Rht-B1b*
JWC 90	Chikugoizumi	Breeders line	Modern	Fukuoka	Kyushu	II	*Rht-D1a*	*Rht-B1b*
JWC 91	Komugi Norin 20	Breeders line	Modern	Saga	Kyushu	III	*Rht-D1a*	*Rht-B1a*
JWC 92	Fujimi komugi	Breeders line	Modern	Saitama	Kanto/Tosan	II	*Rht-D1a*	*Rht-B1b*
JWC 93	Shirasagi komugi	Breeders line	Modern	Hiroshima	Kinki/Chugoku/Shikoku	II	*Rht-D1a*	*Rht-B1b*
JWC 94	Junrei komugi	Breeders line	Modern	Kagawa	Kinki/Chugoku/Shikoku	II	*Rht-D1a*	*Rht-B1b*
JWC 95	Hachiman komugi	Breeders line	Modern	Iwate	Tohoku	I	*Rht-D1b*	*Rht-B1a*
JWC 96	Chinese Spring	Standard line		Sichuan	China	III	*Rht-D1a*	*Rht-B1a*

1) from the NIAS Genebank (http://www.gene.affrc.go.jp/databases-core_collections_jw_en.php)

2) Classical: landraces, pure selected lines, and breeders line before and lines bred before 1920's; Modern: lines bred after 1930's

3) detailed map location for the areas in Kobayashi et al. (2016, Breed Sci, 66:213-225)

4) from this study

5) genorypes for *Rht* genes from Kojima et al. (2017, Bull. NARO Crop Sci., 1:1-13) and this study

Supplemental Table 2. Primer sequences for detecting the alleles of *Rht-B1* and *Rht-D1* *

Alleles	Primer sequences	
	Foreward	Reverse
Rht-B1a (wild type)	5'- GGT AGG GAG GCG AGA GGC GAG -3'	5'- CAT CCC CAT GGC CAT CTC GAG CTG -3'
Rht-B1b (dwarf)	5'- GGT AGG GAG GCG AGA GGC GAG -3'	5'- CAT CCC CAT GGC CAT CTC GAG CTA -3'
Rht-D1a (wild type)	5'- GGC AAG CAA AAG CTT CGC G -3'	5'- GGC CAT CTC GAG CTG CAC -3'
Rht-D1b (dwarf)	5'- CGC GCA ATT ATT GGC CAG AGA TAG -3'	5'- CCC CAT GGC CAT CTC GAG CTG CTA -3'

*from Ellis et al. (2002)

Supplemental Table 3. Summary of mapping statistics in three methods

Accession ID*	amplicon-seq				RAD-seq				GBS				total	
	homozygous sites	alternative homozygous sites	mean depth	% of mapped reads	homozygous sites	alternative homozygous sites	mean depth	% of mapped reads	homozygous sites	alternative homozygous sites	mean depth	% of mapped reads	homozygous sites	alternative homozygous sites
JWC01	476	412	83.60	88.52	5288	5399	7.06	69.01	10004	8638	5.83	71.97	15768	14449
JWC02	453	435	79.15	87.10	5376	5311	10.63	65.27	10330	8312	5.32	72.01	16159	14058
JWC03	481	407	91.88	89.43	5146	5541	8.26	68.90	10080	8562	5.29	70.34	15707	14510
JWC04	485	403	92.88	90.20	5662	5025	4.13	65.26	10502	8140	4.94	70.23	16649	13568
JWC05	693	195	79.60	90.14	9259	1428	12.85	59.62	15344	3298	4.79	73.28	25296	4921
JWC06	666	222	89.28	89.98	8826	1861	8.62	62.33	15021	3621	4.89	73.54	24513	5704
JWC07	641	247	92.58	90.14	8723	1964	10.97	60.24	14541	4101	4.81	72.20	23905	6312
JWC08	665	223	82.43	88.99	8602	2085	11.03	60.86	14665	3977	4.93	73.10	23932	6285
JWC09	686	202	99.54	90.84	8572	2115	10.77	61.21	14708	3934	5.05	73.29	23966	6251
JWC10	687	201	101.20	90.75	9347	1340	14.71	60.66	15284	3358	4.74	72.31	25318	4899
JWC11	591	297	85.76	89.52	7338	3349	14.43	60.44	12952	5690	4.53	70.69	20881	9336
JWC12	654	234	88.40	89.96	8255	2432	3.39	63.75	14325	4317	4.90	71.93	23234	6983
JWC13	681	207	99.31	90.66	8915	1772	11.99	60.65	15120	3522	4.67	73.26	24716	5501
JWC14	659	229	85.55	90.32	8387	2300	11.67	61.20	14395	4247	4.71	71.66	23441	6776
JWC15	665	223	97.62	90.93	8519	2168	12.11	61.17	14525	4117	3.07	71.77	23709	6508
JWC16	707	181	95.57	91.46	8527	2160	4.25	64.48	14595	4047	4.60	73.30	23829	6388
JWC17	635	253	96.19	90.41	7925	2762	10.82	62.77	13723	4919	4.47	72.57	22283	7934
JWC18	664	224	98.69	90.58	8514	2173	8.53	65.11	14507	4135	4.51	72.43	23685	6532
JWC19	676	212	88.73	90.60	8413	2274	8.91	66.39	14528	4114	4.57	71.67	23617	6600
JWC20	542	346	96.01	90.16	7818	2869	12.77	61.40	13705	4937	4.51	71.71	22065	8152
JWC21	676	212	93.27	90.50	8979	1708	12.31	60.37	14979	3663	4.68	72.75	24634	5583
JWC22	634	254	85.66	89.88	8253	2434	13.49	59.83	13960	4682	4.88	71.17	22847	7370
JWC23	678	210	89.00	90.10	8622	2065	11.47	61.85	14780	3862	4.51	71.95	24080	6137
JWC24	680	208	66.51	86.11	8940	1747	12.62	58.81	14954	3688	3.02	71.68	24574	5643
JWC25	688	200	62.02	84.79	8589	2098	10.79	61.19	14668	3974	3.11	71.56	23945	6272
JWC26	632	256	51.85	81.80	8468	2219	14.57	58.96	14519	4123	2.47	71.45	23619	6598
JWC27	675	213	93.35	89.13	8994	1693	12.38	61.70	14979	3663	5.34	72.59	24648	5569
JWC28	664	224	96.09	90.50	8513	2174	16.47	59.85	14509	4133	4.90	72.36	23686	6531
JWC29	690	198	92.78	89.81	8620	2067	13.35	60.37	14643	3999	4.38	69.22	23953	6264
JWC30	694	194	88.40	90.27	8842	1845	3.56	63.06	14925	3717	5.00	72.82	24461	5756
JWC31	677	211	79.25	90.17	8642	2045	4.65	63.43	15016	3626	4.83	73.62	24335	5882
JWC32	676	212	106.82	90.27	8786	1901	12.58	60.28	14854	3788	5.00	71.90	24316	5901

JWC33	673	215	76.02	86.75	9356	1331	6.97	67.01	15147	3495	10.98	66.36	25176	5041
JWC34	702	186	89.59	87.87	8919	1768	11.59	60.95	14887	3755	13.97	66.66	24508	5709
JWC35	652	236	90.52	87.61	8639	2048	2.84	60.64	14112	4530	15.60	65.62	23403	6814
JWC36	675	213	90.29	87.81	9005	1682	11.00	61.91	14971	3671	15.37	66.50	24651	5566
JWC37	679	209	92.23	89.73	9092	1595	12.53	60.58	15039	3603	4.87	71.87	24810	5407
JWC38	672	216	78.59	91.25	9365	1322	11.17	61.74	15146	3496	4.74	72.37	25183	5034
JWC39	626	262	93.71	90.20	8144	2543	10.56	61.43	13818	4824	4.30	69.92	22588	7629
JWC40	698	190	95.39	90.15	9366	1321	12.08	60.08	15414	3228	4.40	72.95	25478	4739
JWC41	665	223	106.93	91.10	8139	2548	9.22	65.74	14265	4377	4.35	72.72	23069	7148
JWC42	657	231	104.12	90.73	8989	1698	13.12	59.47	14773	3869	4.47	72.29	24419	5798
JWC43	678	210	90.98	88.20	9219	1468	14.82	60.89	15374	3268	4.69	72.40	25271	4946
JWC44	701	187	95.67	89.51	8816	1871	11.12	61.31	14860	3782	4.81	72.66	24377	5840
JWC45	691	197	89.69	89.09	8569	2118	5.85	58.05	14642	4000	4.57	72.65	23902	6315
JWC46	426	462	92.57	89.33	5004	5683	12.13	61.98	9624	9018	4.52	69.55	15054	15163
JWC47	568	320	99.25	90.08	7748	2939	10.34	62.27	13136	5506	4.65	66.42	21452	8765
JWC48	528	360	108.51	90.78	6242	4445	12.56	61.49	11808	6834	4.86	70.85	18578	11639
JWC49	549	339	105.96	90.52	7606	3081	8.18	65.18	13153	5489	4.23	69.91	21308	8909
JWC50	546	342	96.96	90.06	7536	3151	7.42	63.49	13377	5265	3.94	70.32	21459	8758
JWC51	518	370	98.70	90.02	7679	3008	13.81	60.58	13155	5487	3.91	69.78	21352	8865
JWC52	515	373	96.84	89.96	6913	3774	14.23	61.93	12201	6441	4.01	70.75	19629	10588
JWC53	507	381	97.59	89.22	7530	3157	14.59	59.73	12972	5670	3.85	69.68	21009	9208
JWC54	565	323	81.42	88.73	7022	3665	13.24	60.70	12700	5942	4.22	72.01	20287	9930
JWC55	514	374	101.75	89.90	5597	5090	3.04	67.31	10802	7840	4.69	68.34	16913	13304
JWC56	642	246	87.95	89.07	8666	2021	9.32	61.37	14275	4367	4.98	70.98	23583	6634
JWC57	564	324	108.00	90.54	7215	3472	4.50	62.20	12734	5908	4.41	71.94	20513	9704
JWC58	560	328	107.83	90.16	7444	3243	14.47	60.00	12655	5987	4.03	71.16	20659	9558
JWC59	542	346	91.18	89.15	7810	2877	13.05	61.10	13713	4929	4.20	71.24	22065	8152
JWC60	468	420	100.74	90.12	6579	4108	15.27	62.51	11410	7232	4.63	70.25	18457	11760
JWC61	529	359	81.24	88.95	6138	4549	15.04	61.57	11602	7040	4.61	71.85	18269	11948
JWC62	541	347	88.87	88.78	6802	3885	10.51	61.42	12100	6542	3.91	68.49	19443	10774
JWC63	620	268	95.85	89.80	8588	2099	3.67	64.15	14386	4256	2.90	70.79	23594	6623
JWC64	598	290	93.70	90.19	8232	2455	11.34	61.22	14093	4549	3.22	40.12	22923	7294
JWC65	536	352	105.68	89.31	7147	3540	11.61	63.54	12099	6543	3.59	55.20	19782	10435
JWC66	627	261	86.36	90.06	8222	2465	12.14	60.13	14185	4457	3.24	43.69	23034	7183
JWC67	534	354	83.69	88.09	6380	4307	14.00	61.62	11718	6924	3.89	68.69	18632	11585
JWC68	497	391	84.64	89.01	5541	5146	4.08	67.88	10882	7760	4.35	70.34	16920	13297
JWC69	457	431	83.95	88.19	5480	5207	15.95	61.51	10078	8564	4.01	69.55	16015	14202

JWC70	465	423	84.18	88.57	5795	4892	11.72	63.07	10722	7920	4.05	69.24	16982	13235
JWC71	493	395	71.49	86.99	6039	4648	11.78	62.43	11315	7327	4.13	70.48	17847	12370
JWC72	458	430	91.52	88.94	5738	4949	11.57	62.91	10956	7686	3.68	69.19	17152	13065
JWC73	515	373	96.30	89.47	5907	4780	14.15	61.93	11118	7524	4.39	69.64	17540	12677
JWC74	564	324	80.40	87.66	7901	2786	5.61	63.33	13252	5390	3.51	69.91	21717	8500
JWC75	525	363	88.32	89.87	6589	4098	12.16	65.31	11669	6973	4.49	70.92	18783	11434
JWC76	529	359	80.94	90.04	6910	3777	9.87	64.12	12522	6120	4.60	71.45	19961	10256
JWC77	527	361	76.44	87.48	5961	4726	13.12	62.63	11199	7443	4.14	70.81	17687	12530
JWC78	482	406	80.64	87.81	6175	4512	14.77	62.76	11057	7585	4.31	68.55	17714	12503
JWC79	578	310	86.14	89.94	7664	3023	11.70	62.33	13709	4933	4.35	70.20	21951	8266
JWC80	598	290	85.30	89.06	7678	3009	9.91	62.33	13265	5377	3.76	68.87	21541	8676
JWC81	588	300	90.94	88.85	8140	2547	14.37	60.10	13972	4670	4.02	71.00	22700	7517
JWC82	448	440	89.83	88.94	5436	5251	12.00	61.94	10767	7875	4.12	69.16	16651	13566
JWC83	471	417	91.26	89.51	5602	5085	12.99	63.10	10989	7653	4.45	71.17	17062	13155
JWC84	527	361	87.92	90.09	6594	4093	11.95	64.07	12052	6590	4.48	71.18	19173	11044
JWC85	440	448	85.29	88.08	5958	4729	15.85	63.82	10936	7706	4.30	70.11	17334	12883
JWC86	499	389	80.00	89.46	6463	4224	4.66	65.14	11782	6860	4.65	70.45	18744	11473
JWC87	582	306	82.66	89.53	7473	3214	11.87	62.10	12941	5701	4.20	70.56	20996	9221
JWC88	553	335	76.06	89.72	7704	2983	9.53	62.31	13645	4997	3.92	70.39	21902	8315
JWC89	589	299	96.17	89.67	8276	2411	11.88	61.29	13969	4673	4.25	71.88	22834	7383
JWC90	536	352	98.01	89.98	7760	2927	11.63	62.47	13477	5165	3.98	70.11	21773	8444
JWC91	625	263	91.86	89.85	8131	2556	7.82	66.10	13814	4828	4.82	66.36	22570	7647
JWC92	524	364	88.58	88.97	7642	3045	12.04	61.12	13174	5468	4.56	68.37	21340	8877
JWC93	542	346	90.02	89.15	7814	2873	11.88	61.36	13707	4935	3.78	70.91	22063	8154
JWC94	560	328	82.46	87.88	8423	2264	9.65	61.92	14180	4462	4.16	70.69	23163	7054
JWC95	529	359	79.53	88.04	7708	2979	11.14	63.02	18427	215	4.13	73.04	26664	3553
JWC96	882	6	79.13	89.17	10196	491	5.32	64.01	12039	6603	4.77	71.24	23117	7100

*: from the NIAS Genebank (http://www.gene.affrc.go.jp/databases-core_collections_jw_en.php)

Supplemental Table 4. SNP marker distribution in the A, B, D and whole genomes

Chr.	No. of SNP markers				SNP marker density (Mb/marker)*			
	A	B	D	Total	A	B	D	Total
1	1906	2070	350	4326	0.314	0.338	1.425	0.416
2	1978	2728	651	5357	0.398	0.298	1.009	0.421
3	1282	3362	295	4939	0.588	0.253	2.100	0.451
4	1787	748	131	2666	0.422	0.901	3.957	0.730
5	1460	2086	213	3759	0.489	0.343	2.676	0.532
6	1479	2756	278	4513	0.421	0.265	1.782	0.410
7	2028	2206	364	4598	0.367	0.346	1.766	0.468
unknown				59				5.959
All	11920	15956	2282	30217	0.417	0.329	1.753	0.482

Chr., chromosome

*The length of each chromosome was referred to Zhu et al. (2021)

Supplemental Table 5. Pairwise mean Fst values (Weir and Coclerham) between three populations calculated SNPs between populations

Population	Population	Weir and Cockerham mean Fst
I	II	0.220
I	III	0.268
II	III	0.151

Supplemental Table 6. Marker–trait associations (MTAs) over multiple years for the days to heading (DH)

Chr.	Position	Marker	Year	log10(-p)	Marker R^2
4D	497,933,062	G_Chr4D_497933062	2018	4.216	0.179
			2017	3.416	0.129
			2020	3.122	0.112
6B	35,332,901	G_Chr6B_35332901	2019	3.814	0.148
			2018	3.667	0.150
	35,332,935	G_Chr6B_35332935	2019	3.814	0.148
			2018	3.667	0.150
	35,332,964	G_Chr6B_35332964	2019	3.814	0.148
			2018	3.667	0.150
	35,465,452	G_Chr6B_35465452	2019	3.814	0.148
			2018	3.667	0.150
	35,612,523	G_Chr6B_35612523	2019	3.607	0.138
			2018	3.497	0.142
	35,633,064	G_Chr6B_35633064	2019	3.607	0.138
			2018	3.497	0.142
	35,633,083	G_Chr6B_35633083	2019	3.814	0.148
			2018	3.667	0.150
	35,858,833	G_Chr6B_35858833	2019	3.607	0.138
			2018	3.497	0.142
	35,859,846	G_Chr6B_35859846	2019	3.814	0.148
			2018	3.667	0.150
	36,569,353	G_Chr6B_36569353	2019	3.393	0.128
			2016	3.058	0.112
	36,600,945	G_Chr6B_36600945	2019	4.013	0.158
			2017	3.402	0.129
			2018	3.300	0.132
			2016	3.227	0.119
	36,600,970	G_Chr6B_36600970	2018	3.643	0.149
			2016	3.270	0.121
			2019	3.016	0.111
6D	130,680,696	G_Chr6D_130680696	2017	3.909	0.153
			2020	3.642	0.136
			2019	3.386	0.128
			2018	3.225	0.128
	133,764,781	R_Chr6D_133764781	2017	3.559	0.136
			2020	3.241	0.118
			2019	3.050	0.112
			2018	3.042	0.119
	300,930,272	G_Chr6D_300930272	2017	3.464	0.132
			2020	3.271	0.119
			2019	3.255	0.122

Supplemental Table 7. Marker–trait associations (MTAs) detected over multiple years for the culm length (CL)

Chr.	Position	Marker	Year	log10(-p)	Marker R^2
2D	22,056,470	G_Chr2D_22056470	2020	3.775	0.162
			2018	3.570	0.151
	22,056,546	G_Chr2D_22056546	2020	3.775	0.162
			2018	3.570	0.151
	22,106,696	G_Chr2D_22106696	2020	3.775	0.162
			2018	3.570	0.151
	22,106,742	G_Chr2D_22106742	2020	3.775	0.162
			2018	3.570	0.151
	23,050,974	G_Chr2D_23050974	2020	3.775	0.162
			2018	3.570	0.151
	23,050,984	G_Chr2D_23050984	2020	3.775	0.162
			2018	3.570	0.151
	23,051,001	G_Chr2D_23051001	2020	3.775	0.162
			2018	3.570	0.151
	23,070,511	G_Chr2D_23070511	2020	3.775	0.162
			2018	3.570	0.151
	23,071,570	G_Chr2D_23071570	2020	3.775	0.162
			2018	3.570	0.151
	23,071,583	G_Chr2D_23071583	2020	3.775	0.162
			2018	3.570	0.151
	23,071,624	G_Chr2D_23071624	2020	3.775	0.162
			2018	3.570	0.151
	23,698,547	G_Chr2D_23698547	2020	3.775	0.162
			2018	3.570	0.151
	23,849,102	G_Chr2D_23849102	2020	3.650	0.155
			2018	3.330	0.138
	23,849,108	G_Chr2D_23849108	2020	3.650	0.155
			2018	3.330	0.138
	23,849,120	G_Chr2D_23849120	2020	3.650	0.155
			2018	3.330	0.138
	23,860,361	G_Chr2D_23860361	2020	3.650	0.155
			2018	3.330	0.138
	23,860,412	G_Chr2D_23860412	2020	3.650	0.155
			2018	3.330	0.138
	23,860,424	G_Chr2D_23860424	2020	3.650	0.155
			2018	3.330	0.138
	23,860,716	G_Chr2D_23860716	2020	3.650	0.155
			2018	3.330	0.138
	23,860,723	G_Chr2D_23860723	2020	3.650	0.155
			2018	3.330	0.138
	23,860,740	G_Chr2D_23860740	2020	3.650	0.155
			2018	3.330	0.138
	23,935,165	G_Chr2D_23935165	2020	3.650	0.155
			2018	3.330	0.138
	23,963,647	R_Chr2D_23963647	2020	3.650	0.155
			2018	3.330	0.138
	24,024,355	G_Chr2D_24024355	2020	3.650	0.155
			2018	3.330	0.138
	24,029,297	G_Chr2D_24029297	2020	3.650	0.155
			2018	3.330	0.138
	24,046,218	G_Chr2D_24046218	2020	3.650	0.155
			2018	3.330	0.138

	73,102,274	G_Chr2D_73102274	2019	3.423	0.142
			2018	3.173	0.130
	73,105,801	G_Chr2D_73105801	2019	3.423	0.142
			2018	3.173	0.130
	73,815,962	G_Chr2D_73815962	2019	3.423	0.142
			2018	3.173	0.130
	73,815,974	G_Chr2D_73815974	2019	3.423	0.142
			2018	3.173	0.130
5A	7,337,076	G_Chr5A_7337076	2019	3.575	0.150
			2016	3.325	0.135
			2020	3.289	0.136
			2018	3.132	0.128
	9,857,051	G_Chr5A_9857051	2018	4.857	0.222
			2019	4.187	0.183
			2020	4.117	0.180
			2016	3.227	0.130
	11,895,571	snp3197_1	2018	3.306	0.137
			2019	3.057	0.124
	12,220,141	R_Chr5A_12220141	2018	3.306	0.137
			2019	3.057	0.124
	439,765,378	R_Chr5A_439765378	2019	3.449	0.144
			2018	3.220	0.133
	439,765,655	R_Chr5A_439765655	2019	3.449	0.144
			2018	3.220	0.133
	439,954,753	G_Chr5A_439954753	2019	3.449	0.144
			2018	3.220	0.133
	439,954,764	G_Chr5A_439954764	2019	3.449	0.144
			2018	3.220	0.133
	439,954,816	G_Chr5A_439954816	2019	3.449	0.144
			2018	3.220	0.133
	440,147,925	R_Chr5A_440147925	2019	3.449	0.144
			2018	3.220	0.133
	440,148,043	R_Chr5A_440148043	2019	3.449	0.144
			2018	3.220	0.133
	440,148,140	R_Chr5A_440148140	2019	3.449	0.144
			2018	3.220	0.133
	440,249,756	R_Chr5A_440249756	2019	3.449	0.144
			2018	3.220	0.133
	440,249,799	R_Chr5A_440249799	2019	3.449	0.144
			2018	3.220	0.133
	440,249,853	R_Chr5A_440249853	2019	3.449	0.144
			2018	3.220	0.133
	440,406,043	G_Chr5A_440406043	2019	3.449	0.144
			2018	3.220	0.133
	440,426,536	G_Chr5A_440426536	2019	3.449	0.144
			2018	3.220	0.133
	440,426,580	G_Chr5A_440426580	2019	3.449	0.144
			2018	3.220	0.133
	440,819,271	G_Chr5A_440819271	2019	3.449	0.144
			2018	3.220	0.133
	442,100,000	R_Chr5A_442100000	2019	3.503	0.146
			2016	3.096	0.123
	665,410,239	G_Chr5A_665410239	2018	4.693	0.212
			2017	3.462	0.146
			2019	3.416	0.142
			2020	3.153	0.129

	666,602,459	G_Chr5A_666602459	2018	4.693	0.212
			2017	3.462	0.146
			2019	3.416	0.142
			2020	3.153	0.129
	708,796,936	G_Chr5A_708796936	2020	3.427	0.143
			2018	3.416	0.143
5B	420,419,443	R_Chr5B_420419443	2017	3.075	0.126
			2018	3.029	0.123
	420,419,473	R_Chr5B_420419473	2017	3.075	0.126
			2018	3.029	0.123
	422,158,046	R_Chr5B_422158046	2017	3.075	0.126
			2018	3.029	0.123
5D	552,393,964	G_Chr5D_552393964	2018	3.221	0.133
			2017	3.165	0.130
			2019	3.143	0.128
	552,849,649	G_Chr5D_552849649	2018	3.221	0.133
			2017	3.165	0.130
			2019	3.143	0.128
6A	128,687,482	G_Chr6A_128687482	2018	4.194	0.185
			2017	3.461	0.146
			2019	3.371	0.140
	138,855,000	G_Chr6A_138855000	2018	4.194	0.185
			2017	3.461	0.146
			2019	3.371	0.140
	609,939,415	G_Chr6A_609939415	2018	3.502	0.147
			2019	3.253	0.134
			2017	3.168	0.130
	609,939,438	G_Chr6A_609939438	2018	3.502	0.147
			2019	3.253	0.134
			2017	3.168	0.130
	609,939,460	G_Chr6A_609939460	2018	3.502	0.147
			2019	3.253	0.134
			2017	3.168	0.130
6B	717,676,609	G_Chr6B_717676609	2019	4.055	0.176
			2016	3.900	0.164
			2018	3.745	0.160
			2020	3.431	0.143
	717,788,119	G_Chr6B_717788119	2019	4.680	0.210
			2018	4.660	0.211
			2020	4.351	0.193
			2016	3.836	0.161
			2017	3.246	0.134
	718,083,564	G_Chr6B_718083564	2019	3.333	0.138
			2016	3.145	0.126
	718,666,921	G_Chr6B_718666921	2019	3.480	0.145
			2018	3.235	0.134
	718,985,803	R_Chr6B_718985803	2019	3.480	0.145
			2018	3.235	0.134
	719,479,762	G_Chr6B_719479762	2019	3.480	0.145
			2018	3.235	0.134
7D	114,214,642	G_Chr7D_114214642	2019	3.243	0.133
			2018	3.180	0.131
chrUn	18,482,772	G_ChrUnknown_18482772	2020	3.775	0.162
			2018	3.570	0.151
	18,482,783	G_ChrUnknown_18482783	2020	3.775	0.162
			2018	3.570	0.151

Supplemental Table 8. Marker–trait associations (MTAs) detected over multiple years for the spike length (SL)

Chr.	Position	Marker	Year	log10(-p)	Marker R^2
2A	594,606,956	R_Chr2A_594606956	2018	3.516	0.149
			2020	3.061	0.126
	596,914,455	R_Chr2A_596914455	2018	3.516	0.149
			2020	3.061	0.126
	604,204,391	R_Chr2A_604204391	2018	3.516	0.149
			2020	3.061	0.126
2B	803,096,394	G_Chr2B_803096394	2020	3.407	0.144
			2018	3.071	0.126
	19,667,354	R_Chr2D_19667354	2020	3.726	0.161
			2018	3.462	0.146
			2019	3.298	0.138
			2017	3.089	0.127
	19,667,575	R_Chr2D_19667575	2020	3.726	0.161
			2018	3.462	0.146
			2019	3.298	0.138
			2017	3.089	0.127
	19,670,544	G_Chr2D_19670544	2020	3.726	0.161
			2018	3.462	0.146
			2019	3.298	0.138
			2017	3.089	0.127
	19,868,310	G_Chr2D_19868310	2020	3.726	0.161
			2018	3.462	0.146
			2019	3.298	0.138
			2017	3.089	0.127
	19,868,322	G_Chr2D_19868322	2020	3.726	0.161
			2018	3.462	0.146
			2019	3.298	0.138
			2017	3.089	0.127
	19,868,386	G_Chr2D_19868386	2020	3.726	0.161
			2018	3.462	0.146
			2019	3.298	0.138
			2017	3.089	0.127
	20,446,572	G_Chr2D_20446572	2018	5.242	0.246
			2020	4.762	0.218
			2017	3.271	0.137
	21,120,861	G_Chr2D_21120861	2018	5.242	0.246
			2020	4.762	0.218
			2017	3.271	0.137
	21,128,584	G_Chr2D_21128584	2018	5.242	0.246
			2020	4.762	0.218
			2017	3.271	0.137
	21,128,621	G_Chr2D_21128621	2018	5.242	0.246
			2020	4.762	0.218
			2017	3.271	0.137
	21,260,160	G_Chr2D_21260160	2018	5.242	0.246
			2020	4.762	0.218
			2017	3.271	0.137
	21,268,060	G_Chr2D_21268060	2018	5.242	0.246
			2020	4.762	0.218
			2017	3.271	0.137

	21,279,081	G_Chr2D_21279081	2018	5.242	0.246
			2020	4.762	0.218
			2017	3.271	0.137
	21,279,086	G_Chr2D_21279086	2018	5.242	0.246
			2020	4.762	0.218
			2017	3.271	0.137
	21,279,100	G_Chr2D_21279100	2018	5.242	0.246
			2020	4.762	0.218
			2017	3.271	0.137
	21,295,292	G_Chr2D_21295292	2018	5.242	0.246
			2020	4.762	0.218
			2017	3.271	0.137
	21,319,179	G_Chr2D_21319179	2018	5.242	0.246
			2020	4.762	0.218
			2017	3.271	0.137
	22,056,470	G_Chr2D_22056470	2018	5.094	0.237
			2020	4.851	0.223
			2019	3.719	0.160
			2017	3.073	0.127
	22,056,546	G_Chr2D_22056546	2018	5.094	0.237
			2020	4.851	0.223
			2019	3.719	0.160
			2017	3.073	0.127
	22,106,696	G_Chr2D_22106696	2018	5.094	0.237
			2020	4.851	0.223
			2019	3.719	0.160
			2017	3.073	0.127
	22,106,742	G_Chr2D_22106742	2018	5.094	0.237
			2020	4.851	0.223
			2019	3.719	0.160
			2017	3.073	0.127
	23,050,974	G_Chr2D_23050974	2018	5.094	0.237
			2020	4.851	0.223
			2019	3.719	0.160
			2017	3.073	0.127
2D	23,050,984	G_Chr2D_23050984	2018	5.094	0.237
			2020	4.851	0.223
			2019	3.719	0.160
			2017	3.073	0.127
	23,051,001	G_Chr2D_23051001	2018	5.094	0.237
			2020	4.851	0.223
			2019	3.719	0.160
			2017	3.073	0.127
	23,070,511	G_Chr2D_23070511	2018	5.094	0.237
			2020	4.851	0.223
			2019	3.719	0.160
			2017	3.073	0.127
	23,071,570	G_Chr2D_23071570	2018	5.094	0.237
			2020	4.851	0.223
			2019	3.719	0.160
			2017	3.073	0.127
	23,071,583	G_Chr2D_23071583	2018	5.094	0.237
			2020	4.851	0.223
			2019	3.719	0.160

		2017	3.073	0.127
23,071,624	G_Chr2D_23071624	2018	5.094	0.237
		2020	4.851	0.223
		2019	3.719	0.160
		2017	3.073	0.127
23,698,547	G_Chr2D_23698547	2018	5.094	0.237
		2020	4.851	0.223
		2019	3.719	0.160
		2017	3.073	0.127
23,758,078	G_Chr2D_23758078	2020	3.996	0.175
		2019	3.479	0.147
		2018	3.068	0.126
23,758,087	G_Chr2D_23758087	2020	3.996	0.175
		2019	3.479	0.147
		2018	3.068	0.126
23,849,102	G_Chr2D_23849102	2018	4.873	0.224
		2020	4.367	0.196
		2019	3.039	0.124
23,849,108	G_Chr2D_23849108	2018	4.873	0.224
		2020	4.367	0.196
		2019	3.039	0.124
23,849,120	G_Chr2D_23849120	2018	4.873	0.224
		2020	4.367	0.196
		2019	3.039	0.124
23,860,361	G_Chr2D_23860361	2018	4.873	0.224
		2020	4.367	0.196
		2019	3.039	0.124
23,860,412	G_Chr2D_23860412	2018	4.873	0.224
		2020	4.367	0.196
		2019	3.039	0.124
23,860,424	G_Chr2D_23860424	2018	4.873	0.224
		2020	4.367	0.196
		2019	3.039	0.124
23,860,716	G_Chr2D_23860716	2018	4.873	0.224
		2020	4.367	0.196
		2019	3.039	0.124
23,860,723	G_Chr2D_23860723	2018	4.873	0.224
		2020	4.367	0.196
		2019	3.039	0.124
23,860,740	G_Chr2D_23860740	2018	4.873	0.224
		2020	4.367	0.196
		2019	3.039	0.124
23,935,165	G_Chr2D_23935165	2018	4.873	0.224
		2020	4.367	0.196
		2019	3.039	0.124
23,963,647	R_Chr2D_23963647	2018	4.873	0.224
		2020	4.367	0.196
		2019	3.039	0.124
24,024,355	G_Chr2D_24024355	2018	4.873	0.224
		2020	4.367	0.196
		2019	3.039	0.124
24,029,297	G_Chr2D_24029297	2018	4.873	0.224
		2020	4.367	0.196
		2019	3.039	0.124

	24,046,218	G_Chr2D_24046218	2018	4.873	0.224
			2020	4.367	0.196
			2019	3.039	0.124
	24,726,982	tarc1446	2018	5.627	0.268
			2020	4.863	0.224
			2017	4.153	0.184
			2019	4.000	0.175
			2016	3.567	0.151
3A	60,189,000	tarc0517	2018	3.827	0.166
			2020	3.376	0.142
	716,812,904	G_Chr3A_716812904	2018	3.278	0.137
			2016	3.177	0.130
			2019	3.106	0.128
3D	576,645,901	G_Chr3D_576645901	2018	4.917	0.227
			2020	4.564	0.207
			2017	4.321	0.194
			2016	3.271	0.135
			2019	3.035	0.124
4A	107,925,196	G_Chr4A_107925196	2018	3.312	0.138
			2020	3.281	0.137
	145,251,348	G_Chr4A_145251348	2018	3.312	0.138
			2020	3.281	0.137
5B	385,614,577	R_Chr5B_385614577	2018	3.593	0.153
			2020	3.386	0.142
			2017	3.094	0.128
	385,636,679	G_Chr5B_385636679	2018	3.593	0.153
			2020	3.386	0.142
			2017	3.094	0.128
7B	99,226,546	G_Chr7B_99226546	2016	3.131	0.128
			2017	3.018	0.124
	100,132,247	G_Chr7B_100132247	2016	3.131	0.128
			2017	3.018	0.124
	100,579,102	R_Chr7B_100579102	2016	3.131	0.128
			2017	3.018	0.124
	100,579,115	R_Chr7B_100579115	2016	3.131	0.128
			2017	3.018	0.124
	100,579,166	R_Chr7B_100579166	2016	3.131	0.128
			2017	3.018	0.124
	100,579,194	R_Chr7B_100579194	2016	3.131	0.128
			2017	3.018	0.124
	203,239,104	G_Chr7B_203239104	2018	3.397	0.143
			2017	3.180	0.132
			2020	3.177	0.132
	712,264,197	G_Chr7B_712264197	2020	4.208	0.187
			2018	3.551	0.151
chrUn	18,482,772	G_ChrUnknown_18482772	2018	5.094	0.237
			2020	4.851	0.223
			2019	3.719	0.160
			2017	3.073	0.127
	18,482,783	G_ChrUnknown_18482783	2018	5.094	0.237
			2020	4.851	0.223
			2019	3.719	0.160
			2017	3.073	0.127

Supplemental Table 9. Marker–trait associations (MTAs) detected over multiple years for the spike density (SD)

Chr.	Position	Marker	Year	log10(-p)	Marker R^2
2D	15,341,074	G_Chr2D_15341074	2019	3.318	0.139
			2017	3.133	0.128
2D	15,341,132	G_Chr2D_15341132	2019	3.318	0.139
			2017	3.133	0.128
2D	19,667,354	R_Chr2D_19667354	2018	3.182	0.132
			2019	3.144	0.130
			2020	3.048	0.122
2D	19,667,575	R_Chr2D_19667575	2018	3.182	0.132
			2019	3.144	0.130
			2020	3.048	0.122
2D	19,670,544	G_Chr2D_19670544	2018	3.182	0.132
			2019	3.144	0.130
			2020	3.048	0.122
2D	19,868,310	G_Chr2D_19868310	2018	3.182	0.132
			2019	3.144	0.130
			2020	3.048	0.122
2D	19,868,322	G_Chr2D_19868322	2018	3.182	0.132
			2019	3.144	0.130
			2020	3.048	0.122
2D	19,868,386	G_Chr2D_19868386	2018	3.182	0.132
			2019	3.144	0.130
			2020	3.048	0.122
2D	20,446,572	G_Chr2D_20446572	2020	3.863	0.165
			2018	3.804	0.165
			2017	3.283	0.136
			2019	3.228	0.134
2D	21,120,861	G_Chr2D_21120861	2020	3.863	0.165
			2018	3.804	0.165
			2017	3.283	0.136
			2019	3.228	0.134
2D	21,128,584	G_Chr2D_21128584	2020	3.863	0.165
			2018	3.804	0.165
			2017	3.283	0.136
			2019	3.228	0.134
2D	21,128,621	G_Chr2D_21128621	2020	3.863	0.165
			2018	3.804	0.165
			2017	3.283	0.136
			2019	3.228	0.134
2D			2020	3.863	0.165

	21,260,160	G_Chr2D_21260160	2018	3.804	0.165
			2017	3.283	0.136
			2019	3.228	0.134
	21,268,060	G_Chr2D_21268060	2020	3.863	0.165
			2018	3.804	0.165
			2017	3.283	0.136
			2019	3.228	0.134
	21,279,081	G_Chr2D_21279081	2020	3.863	0.165
			2018	3.804	0.165
			2017	3.283	0.136
			2019	3.228	0.134
	21,279,086	G_Chr2D_21279086	2020	3.863	0.165
			2018	3.804	0.165
			2017	3.283	0.136
			2019	3.228	0.134
	21,279,100	G_Chr2D_21279100	2020	3.863	0.165
			2018	3.804	0.165
			2017	3.283	0.136
			2019	3.228	0.134
	21,295,292	G_Chr2D_21295292	2020	3.863	0.165
			2018	3.804	0.165
			2017	3.283	0.136
			2019	3.228	0.134
	21,319,179	G_Chr2D_21319179	2020	3.863	0.165
			2018	3.804	0.165
			2017	3.283	0.136
			2019	3.228	0.134
	24,726,982	tarc1446	2018	3.799	0.165
			2016	3.785	0.162
			2020	3.504	0.146
			2019	3.380	0.142
			2017	3.059	0.124
3D	576,645,901	G_Chr3D_576645901	2020	3.690	0.155
			2016	3.674	0.156
			2017	3.622	0.153
			2019	3.483	0.148
4D	35,728,462	G_Chr4D_35728462	2020	4.054	0.175
			2019	3.281	0.137
5B	541,072,981	G_Chr5B_541072981	2019	3.975	0.174
			2020	3.132	0.127
	544,302,059	G_Chr5B_544302059	2019	4.166	0.185
			2020	3.910	0.167
			2016	3.886	0.167

			2017	3.111	0.127
	544,733,353	G_Chr5B_544733353	2019	4.166	0.185
			2020	3.910	0.167
			2016	3.886	0.167
			2017	3.111	0.127
6A	174,924,572	R_Chr6A_174924572	2016	3.211	0.132
			2019	3.024	0.124
	190,309,836	R_Chr6A_190309836	2018	3.423	0.145
			2016	3.091	0.126
			2019	3.023	0.124
	192,673,544	R_Chr6A_192673544	2018	3.217	0.134
			2016	3.080	0.125
7B	3,469,660	G_Chr7B_3469660	2016	3.810	0.163
			2018	3.080	0.127
	99,226,546	G_Chr7B_99226546	2019	3.257	0.136
			2017	3.152	0.129
	100,132,247	G_Chr7B_100132247	2019	3.257	0.136
			2017	3.152	0.129
	100,579,102	R_Chr7B_100579102	2019	3.257	0.136
			2017	3.152	0.129
	100,579,115	R_Chr7B_100579115	2019	3.257	0.136
			2017	3.152	0.129
	100,579,166	R_Chr7B_100579166	2019	3.257	0.136
			2017	3.152	0.129
	100,579,194	R_Chr7B_100579194	2019	3.257	0.136
			2017	3.152	0.129
7D	31,636,771	G_Chr7D_31636771	2020	3.197	0.130
			2019	3.084	0.127
			2016	3.005	0.122

Supplemental Table 10. Marker–trait associations (MTAs) detected over multiple years for the grain numbers per spike (GNS)

Chr.	Position	Marker	Year	log10(-p)	Marker R^2
2B	29505126	G_Chr2B_29505126	2016	3.721	0.160
			2017	3.467	0.146
			2019	3.445	0.141
			2020	3.035	0.123
	728671668	G_Chr2B_728671668	2017	4.609	0.208
			2018	3.775	0.163
			2020	3.467	0.145
3A	668364722	G_Chr3A_668364722	2020	3.117	0.127
			2017	3.100	0.127
	668364729	G_Chr3A_668364729	2020	3.117	0.127
			2017	3.100	0.127
6B	728450934	G_Chr6B_728450934	2020	3.886	0.167
			2016	3.381	0.142
			2019	3.201	0.129
	730329182	G_Chr6B_730329182	2020	3.939	0.170
			2019	3.932	0.166
			2016	3.046	0.124

Supplemental Table 11. Marker–trait associations (MTAs) detected over multiple years for the grain numbers per spikelet(GNSL)

Chr.	Position	Marker	Year	log10(-p)	Marker R^2
2B	728,671,668	G_Chr2B_728671668	2020	3.590	0.150
			2018	3.125	0.127
			2017	3.086	0.114
7B	711,853,721	G_Chr7B_711853721	2019	3.223	0.130
			2017	3.076	0.113

Supplemental Table 12. Marker–trait associations (MTAs) detected over multiple years for the thousand-kernel weight (TKW)

Chr.	Position	Marker	Year	log10(-p)	Marker R^2
3A	533,585,802	G_Chr3A_533585802	2018	3.284	0.137
			2017	3.032	0.121
	535,032,606	R_Chr3A_535032606	2018	3.284	0.137
			2017	3.032	0.121
	535,032,633	R_Chr3A_535032633	2018	3.284	0.137
			2017	3.032	0.121
	536,261,781	R_Chr3A_536261781	2018	3.284	0.137
			2017	3.032	0.121
	536,295,152	G_Chr3A_536295152	2018	3.284	0.137
			2017	3.032	0.121
	536,666,153	G_Chr3A_536666153	2018	3.284	0.137
			2017	3.032	0.121
6A	523,909,250	G_Chr6A_523909250	2019	3.331	0.138
			2017	3.127	0.126
	524,053,443	R_Chr6A_524053443	2019	3.331	0.138
			2017	3.127	0.126
	524,203,903	R_Chr6A_524203903	2019	3.331	0.138
			2017	3.127	0.126
	524,683,741	R_Chr6A_524683741	2019	3.331	0.138
			2017	3.127	0.126
	524,789,877	G_Chr6A_524789877	2019	3.331	0.138
			2017	3.127	0.126
	525,558,660	G_Chr6A_525558660	2019	3.331	0.138
			2017	3.127	0.126
	525,718,380	R_Chr6A_525718380	2019	3.331	0.138
			2017	3.127	0.126
	525,725,661	R_Chr6A_525725661	2019	3.331	0.138
			2017	3.127	0.126
	525,725,681	R_Chr6A_525725681	2019	3.331	0.138
			2017	3.127	0.126
	525,809,715	R_Chr6A_525809715	2019	3.331	0.138
			2017	3.127	0.126
	525,809,725	R_Chr6A_525809725	2019	3.331	0.138
			2017	3.127	0.126
	525,809,776	R_Chr6A_525809776	2019	3.331	0.138
			2017	3.127	0.126
	526,638,684	G_Chr6A_526638684	2019	3.331	0.138
			2017	3.127	0.126
	535,968,276	G_Chr6A_535968276	2019	3.710	0.158
			2016	3.455	0.144
	537,981,999	R_Chr6A_537981999	2019	3.710	0.158
			2016	3.455	0.144
6D	485,252,487	G_Chr6D_485252487	2016	3.375	0.140
			2019	3.095	0.126
